# Mitochondria-targeted oligomeric α-synuclein induces TOM40 degradation and mitochondrial dysfunction in Parkinson’s disease and parkinsonism-dementia of Guam

**DOI:** 10.1038/s41419-024-07258-5

**Published:** 2024-12-18

**Authors:** Velmarini Vasquez, Manohar Kodavati, Joy Mitra, Indira Vedula, Dale J. Hamilton, Ralph M. Garruto, K. S. Rao, Muralidhar L. Hegde

**Affiliations:** 1 Division of DNA Repair Research, Center for Neuroregeneration, Department of Neurosurgery, Houston Methodist Research Institute, Houston, TX USA; 2 Neuroscience Center, Instituto de Investigaciones Científicas y Servicios de Alta Tecnología, (INDICASAT AIP), Panama City, Panama; 3 Center for Bioenergetics, Houston Methodist Research Institute, Houston, TX USA; 4 Department of Medicine, Houston Methodist, Weill Cornell Medicine affiliate, Houston, TX USA; 5 Departments of Anthropology and Biological Sciences, Binghamton University, State University of New York, Binghamton, NY USA; 6 Department of Biotechnology, KLEF Deemed to be University, Vaddeswaram, India; 7 Department of Neuroscience, Weill Cornell Medical College, New York, NY USA

**Keywords:** Cell death in the nervous system, Molecular neuroscience

## Abstract

Mitochondrial dysfunction is a central aspect of Parkinson’s disease (PD) pathology, yet the underlying mechanisms are not fully understood. This study investigates the link between α-Synuclein (α-Syn) pathology and the loss of translocase of the outer mitochondrial membrane 40 (TOM40), unraveling its implications for mitochondrial dysfunctions in neurons. We discovered that TOM40 protein depletion occurs in the brains of patients with Guam Parkinsonism-Dementia (Guam PD) and cultured neurons expressing α-Syn proteinopathy, notably, without corresponding changes in TOM40 mRNA levels. Cultured neurons expressing α-Syn mutants, with or without a mitochondria-targeting signal (MTS) underscores the role of α-Syn’s mitochondrial localization in inducing TOM40 degradation. PDe-related etiological factors, such as 6-hydroxydopamine or ROS/metal ions stress, which promotes α-Syn oligomerization, exacerbate TOM40 depletion in PD patient-derived cells with SNCA gene triplication. Although α-Syn interacts with both TOM40 and TOM20 in the outer mitochondrial membrane, degradation is selective for TOM40, which occurs via the ubiquitin-proteasome system (UPS) pathway. Our comprehensive analyses using Seahorse technology, mitochondrial DNA sequencing, and damage assessments, demonstrate that mutant α-Syn-induced TOM40 loss results in mitochondrial dysfunction, characterized by reduced membrane potential, accumulation of mtDNA damage, deletion/insertion mutations, and altered oxygen consumption rates. Notably, ectopic supplementation of TOM40 or reducing pathological forms of α-Syn using ADP-ribosylation inhibitors ameliorate these mitochondrial defects, suggesting potential therapeutic avenues. In conclusion, our findings provide crucial mechanistic insights into how α-Syn accumulation leads to TOM40 degradation and mitochondrial dysfunction, offering insights for targeted interventions to alleviate mitochondrial defects in PD.

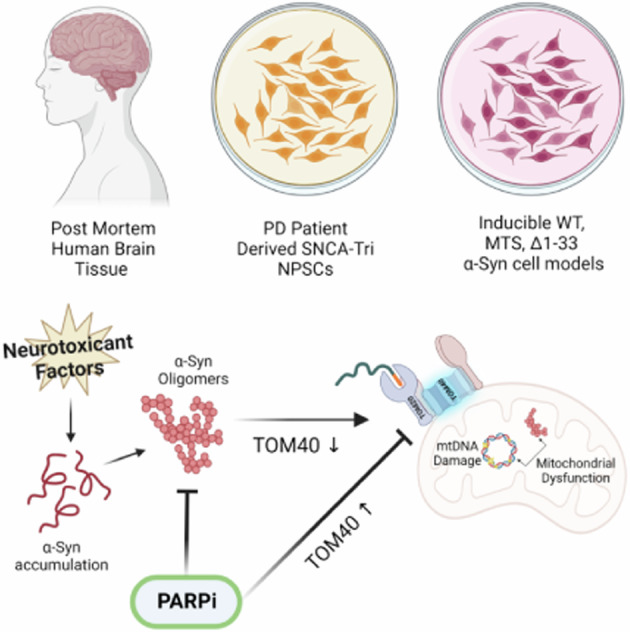

## Introduction

Parkinson’s disease (PD) is characterized by the accumulation of alpha-synuclein (α-Syn) protein aggregates and mitochondrial dysfunction in vulnerable dopaminergic neurons [1, 2]. Despite significant research, the sequence and nature of the bidirectional interaction between α-Syn accumulation and mitochondrial dysfunction are not fully understood. Emerging evidence suggests that α-Syn’s translocation into mitochondria disrupts key mitochondrial processes observed in PD patients [2–4]. For instance, dopaminergic neurons overexpressing mutant A53T α-Syn exhibit mitochondrial complex I deficiency akin to that seen in the substantia nigra of PD patients, resulting in the overproduction of mitochondrial reactive oxygen species (mtROS) [5–7]. Potential pathological consequences of mtROS build-up include mitochondrial DNA (mtDNA) damage, a signature of mitochondrial dysfunction in PD [8–10]. We and others have demonstrated that α-Syn expression induces strand breaks in the neuronal genome and its misfolding and oligomerization heighten its DNA-nicking activity [11, 12]. However, the exact mechanism by which mtDNA damage accrues in association with α-Syn aggregation in PD-affected neurons remains largely unexplored.

The translocases of the outer mitochondrial membrane (TOM) complex, crucial for the import of more than 90% of the 1136 nuclear-encoded proteins that compose the mitochondrial proteome, are important for maintaining normal organelle physiology [13–16]. The TOM complex consists of the channel-forming β-barrel protein TOM40, three receptor proteins (TOM20, TOM22, and TOM70), and three small regulatory subunits [17]. The import process involves TOM20 recognizing the precursor protein mitochondrial targeting sequence, coordinating with TOM22 or TOM70, and cytosolic chaperones from the Hsp90 and Hsp70 classes [18]. The preprotein then traverses the TOM40 channel, reaching the translocase of the inner mitochondrial membrane (TIM23), essential for subsequent cleavage and maturation processes [18]. Notably, among the seven components of the TOM complex, TOM40 stands out as the sole essential component for cell viability and is responsible for translocating intermembrane space proteins without requiring the TOM receptor domains [19–21]. Consequently, any deficit in TOM40 adversely affects preprotein import, disrupting mitochondrial homeostasis and neuronal viability.

TOM40 polymorphisms have been associated with an increased risk of Alzheimer’s disease in different populations, and changes in non-pathological aging-related factors, like changes in brain integrity, body mass index, and cognitive function [22–25]. In the context of PD, while TOM40 single nucleotide polymorphisms (SNPs) like TOM40 ‘523’ may not predict Parkinson’s disease risk, it could serve as a genetic marker for the age of symptom onset in PD [26]. However, altered levels or activities of the TOM complex proteins are evident in human brain tissues from PD patients and relevant animal and cellular models [27–29]. Specifically, α-Syn accumulation is linked to reduced TOM40 protein levels and impaired interaction between TOM20 and TOM22 [27, 28]. Despite ample evidence linking TOM40 dysregulation to α-Syn accumulation in PD, the cellular pathways responsible for this dysregulation remain unclear.

The inhibition of Poly (ADP-ribose) polymerase (PARP), a nuclear enzyme involved in DNA repair, has emerged as a promising therapeutic avenue to reduce inflammation and oxidative stress, both of which are implicated in the pathogenesis of neurodegenerative disorders [30]. In conditions associated with α-Syn toxicity, increased DNA damage activate PARP excessively, leading to the overproduction of poly(ADP-ribose) (PAR) chains. This heightened PARylation does not only impact cellular energy balance and genomic stability but also accelerates α-Syn aggregation [31, 32]. Recent studies have established that PARP inhibition reduce the formation of α-Syn aggregates, potentially through the modulation of autophagy and the clearance of misfolded proteins [31, 33]. By mitigating α-Syn aggregation, PARP inhibition may contribute to reducing neurotoxicity associated with these aggregates.

Here we investigated the regulatory mechanisms by which the presence of α-Syn influences TOM40 protein degradation and its connection with mtDNA damage. We used Guam Parkinsonism-Dementia (Guam PD) and Guam Amyotrophic Lateral Sclerosis (Guam ALS) and Guam non-neurological Control (Guam Control) postmortem brain tissues and stable cell lines expressing different α-Syn variants. Our findings reveal a unique aspect of α-Syn’s impact on mitochondrial function, with pathological, oligomeric forms of α-Syn promoting TOM40 degradation independently of transcriptional changes, as evidenced by stable TOM40 mRNA levels. We also found that while moderate ectopic expression of TOM40 can mitigate the loss of TOM40, ADP-ribosylation inhibitors significantly decreased TOM40 loss while reducing pathological forms of α-Syn using and improving cell survival. Our findings offer vital mechanistic insights into the causal relationship between α-Syn accumulation leading to TOM40 loss, and subsequent mitochondrial dysfunction. These insights hold the potential to inform the development of targeted strategies aimed at alleviating mitochondrial defects in PD.

## Materials and methods

### Protein extraction and immunoblotting

Whole-cell protein extracts were obtained by harvesting cells in ice-cold PBS and lysing them with STEN lysis buffer (50 mM Tris-HCl, pH 7.6; 150 mM NaCl; 0.1% SDS; 1% Nonidet P-40; 2 mM EDTA; and protease inhibitor cocktail) on ice for 20 min. Human brain tissue samples (Guam ALS, Guam PD, and Guam control) were obtained from the Binghamton University Biorepository Archive [34] (Guam ALS, Guam PD, and Guam-control brain tissues, Supplementary Table 1). These samples were obtained as de-identified frozen specimens and were homogenized as we previously described [35].

SDS-PAGE gel electrophoresis was performed according to a standard protocol described [36, 37]. Protein samples for SDS-PAGE were prepared by diluting them with their respective lysis buffers and adding 4× NuPAGE LDS sample buffer. Unless otherwise specified, 20 μg protein was loaded per lane on a NuPAGE 4–12% Bis-Tris gel. Electrophoresis was carried out using NuPAGE MES-SDS running buffer, as described [11]. Membranes were then incubated with primary antibody (Supplementary Table 2) diluted in 1% skim milk in TBS-T for 1 h at RT or overnight at 4 °C. Membranes were washed in 1× TBS-T, then incubated with secondary antibody for 1 h at RT. Detection was performed using WesternSure PREMIUM Chemiluminescent Substrate (Licor, USA).

### Mitochondria protein extraction

Mitochondrial protein isolation was performed using the differential centrifugation method as described in previous studies [38, 39]. All procedures were conducted at 4 °C or on ice to preserve mitochondrial integrity. Cells were first harvested and then homogenized in STM buffer (comprising 250 mM Sucrose, 50 mM Tris-HCl at pH 7.4, 5 mM MgCl_2_, and a protease inhibitor cocktail) using a Glas-Col homogenizer set to 700–1000 rpm. The resulting homogenate was transferred to a centrifuge tube and incubated on ice for 30 min before centrifugation at 800 g for 15 min. The supernatant was collected and subjected to an additional centrifugation step to separate mitochondrial and cytosolic fractions. Further centrifugation of the supernatant at 11,000 × *g* for 15 min yielded a mitochondrial pellet, while the remaining supernatant contained the cytosolic fraction. The mitochondrial pellet was washed and re-centrifuged in STM buffer at 11,000 × *g* for 10 min. Following this, the pellet was suspended in 1× RIPA buffer (50 mM Tris HCl pH 6.8, 1 mM EDTA, 0.5% Triton-X-100, and a protease inhibitor cocktail). The lysed mitochondrial fraction was then centrifuged at 15,700 × *g* for 15 min to remove any residual material.

### Co-immunoprecipitation (Co-IP)

The co-IP of endogenous TOM40 was conducted using protein A/G PLUS agarose beads (Santa Cruz Biotechnology). Cells were harvested and lysed in a buffer containing 0.2% NP-40, 150 mM NaCl, 25 mM Tris-HCl, and 0.1% SDS. The lysate was precleared by incubating it with 1 μg of control IgG antibody along with protein A/G beads to eliminate non-specific binding. After a 30-min incubation at 4 °C, the supernatant was collected and incubated with the TOM40 antibody (Proteintech 18409-1). Subsequently, protein A/G beads were added to the mixture and incubated overnight on a rocker at 4 °C. Following incubation, the antibody-protein-bead complexes were separated by centrifugation at 600 × *g*. The beads were then washed three times to remove any unbound proteins. The bound proteins were then eluted and analyzed by western blotting.

To test the interaction of recombinant α-Syn with TOM proteins, lysates of isolated mitochondria from neural progenitor stem cells (NPSCs) were incubated with 1 µg of recombinant α-Syn in vitro. Co-IP was then performed as above using protein A/G beads and α-Syn antibody.

### Real-time-PCR analysis for mRNA quantitation

Total RNA was extracted using the RNeasy Mini Kit (Qiagen74104, Germany) following the manufacturer’s protocol. For cDNA synthesis, two micrograms of isolated RNA from cells were employed in a 20 μL reaction with the SuperScript III reverse transcriptase kit (Thermo Fisher 18080-044, USA). TOM40, TOM20 and α-Syn in the samples was assessed through SYBR GREEN-based Real-Time-PCR, using the 7000 Real-Time PCR System (Applied Biosystems). The process used SYBR Premix Ex Taq (TaKaRa) and gene-specific primers, which are detailed in Supplementary Table 3. The data were expressed as fold change in mRNA expression relative to the reference samples, set to a baseline value of one. This quantification was determined using the 2^–ΔΔCT^ method [40].

### Human iPSC culture and generation of neural progenitor stem cells (NPSCs)

The control induced pluripotent stem cell (iPSC) line KYOU-DXR0109B (201B7) was obtained from the American Type Culture Collection (ATCC). The cells were cultured at 37 °C and 5% CO_2_ in dishes coated with CellMatrix basement membrane gel (ATCC ACS-3035, USA) and maintained in Essential eight medium (E8M; Thermo Fisher A1517001, USA).

The PD patient-derived iPSC line with α-Syn SNCA gene triplication (SNCA-Tri), designated ND34391*H, was sourced from the CORIELL Institute cell repository. Initially, SNCA-Tri iPSCs were cultivated in 0.1% gelatin-coated 6-well plates with γ-irradiated CF-1 mouse embryonic fibroblasts and DMEM/F12 20% knock-out serum replacement media (Thermo-Fisher 11330-032, 10829018, USA). At passage three, SNCA-Tri iPSCs were transitioned to a feeder-free system, growing in dishes coated with CellMatrix.

NPSCs were derived from both the control and SNCA-Tri iPSC lines using PSC neural induction medium (Thermo Fisher A1647801, USA) following the manufacturer’s instructions and the protocol explained earlier [11].

### Plasmid constructs

Construction of pCW WT-α-Syn-Flag Expression Vector: The doxycycline (Dox) inducible mammalian pCW WT-α-Syn-Flag expression vector was created by inserting the full-length α-Syn, amplified from the pcDNA WT-α-Syn plasmid using DeepVent DNA polymerase (NEB-LABS M0258, USA) and CW WT α-Syn-FLAG F/R primer pair. This fragment was then cloned into the pCW-Cas9 vector (a generous gift from Eric Lander and David Sabatini, Addgene plasmid 50661) at 5´-NheI and 3´-blunt-ended sites. The pCW-Cas9 vector was initially digested with BamHI (NEB R0136, USA), treated with Klenow fragment (DNA polymerase I, large; NEB M0210, USA) to generate a blunt-ended vector backbone, and subsequently digested with NheI (NEB R0131, USA).

Construction of pCW Δ1-33-α-Syn-Flag Expression Vector: The Dox-inducible truncated α-Syn expression vector (pCW-FLAG-Δ1-33-α-Syn) was generated using a similar approach to the WT-α-Syn-Flag vector. The construction utilized the primer pair pCW Δ1-33 α-Syn FLAG F/R.

Construction of Mitochondria-Targeted α-Syn (MTS) Expression Vector: The mitochondria-targeted α-Syn (MTS) expression vector (pCW MTS-α-Syn-Flag expression vector) was produced using a two-step cloning strategy. Initially, a synthetic MTS duplex DNA was created by annealing MTS sense and antisense oligos with sticky ends corresponding to 5´ XbaI and 3´ NcoI sites. Subsequently, the full-length α-Syn coding sequence (cds) was amplified from the pcDNA Syn vector using the Nco F /Sal R primer pair. The pLenti CMV GFP vector (pLenti CMV GFP Blast (659-1), a gift from Eric Campeau and Paul Kaufman, Addgene plasmid # 17445) was digested at XbaI-SalI sites to remove the GFP cds. A tripartite ligation was then performed by combining the pLenti CMV vector backbone, MTS duplex oligo, and α-Syn cds to generate pLenti CMV MTS-α-Syn construct. Finally, MTS-α-Syn cds were amplified using CW MTS-α-Syn forward and reverse primers from this intermediate plasmid pLenti CMV MTS-α-Syn and cloned to the pCW vector, similar to the WT clone. The primer sequences utilized for these constructs are detailed in Supplementary Table 4. All sub-clones and final clones were sequence-verified at least twice before expression in mammalian cell lines.

### Cell culture and treatments

SH-SY5Y neuroblastoma cells were maintained in Dulbecco’s Modified Eagle’s Medium (DMEM) supplemented with 10% fetal bovine serum and 1% penicillin/streptomycin. Transfection of SH-SY5Y cells with Dox-inducible plasmids was carried out using Lipofectamine 2000 (Invitrogen), following the manufacturer’s guidelines. Post-transfection, cells underwent selection against the antibiotic puromycin (InvivoGen, USA) at a concentration of 10 μg/mL. Cells were then cultured in Neurobasal medium (Gibco) supplemented with B-27, 1× Glutamax (Gibco), and 1% penicillin/streptomycin (Corning) containing 10 μM retinoic acid (RA) for seven days to induce neuronal differentiation. To achieve optimal α-Syn expression induction, cells were exposed to Dox at a concentration of 5 μg/mL for varying durations (0, 24, 48, or 72 h), resulting in a substantial two to three-fold increase in total α-Syn levels compared to the baseline.

We evaluated neurotoxicant factors known to promote α-Syn aggregation by treating two cell models: control, or SNCA-Tri NPSCs and α-Syn expressing SH-SY5Y cells. These cells were subjected to a 24-h treatment with media supplemented with either 10 µM Rotenone (Sigma R8875), 10 µM 6-hydroxydopamine hydrobromide (6OHDA, Sigma 162957, USA), 50 µM FeSO_4_ (Sigma F8048), and 50 µM FeCl_3_ (Sigma F7131). Additionally, cells were exposed to 50 nM glucose oxidase (GO) (Sigma G3660-1CAP) for 30 min, followed by media replacement and a one-hour recovery period before cell harvest. To investigate the role of poly (ADP-ribose) polymerase (PARP) in the context of 6OHDA-induced α-Syn aggregation, cells were treated with 10 µM Veliparib, a PARP inhibitor, following the removal of 6OHDA-containing media. This treatment lasted for 24 h before subsequent analyses.

To investigate which protein quality control pathway is involved in TOM40 degradation, we exposed α-Syn expressing SH-SY5Y cells to specific inhibitors targeting these pathways, as illustrated in Fig. 4A. Prior to inducing α-Syn expression, cells were pre-treated for 24 h with the following inhibitors: 1 µM MG132 (Sigma, M8699) to inhibit the UPS pathway; 0.25 nM bafilomycin A (BafA1, Sigma 131793) to increase MDVs accumulation in the cytosol by preventing lysosomal acidification [41]; alternatively the lysosomal protease inhibitors 2.0 µM Pepstatin A (Sigma 516481-M) and 2.0 µM E64d (Sigma E8640) to allow MDVs delivery to the lysosomes but prevent protein degradation [42]; and the mitochondrial division inhibitor 2.0 µM Mdivi-1 (Sigma M0199) to inhibit mitophagy.

### Immunofluorescence (IF)

The pCW-FLAG-α-Syn transduced SH-SY5Y cells were cultured on 8-well chamber slides. For iPSCs and NPSCs, the chamber slides were pre-coated with Matrigel and Geltrex, respectively to facilitate adherence. Fixation of the cells for IF analysis was performed by replacing the media with a 1:1 ratio mixture of fresh media and 8% paraformaldehyde (PFA) in PBS, resulting in a final concentration of 4% PFA. Post-fixation, the slides were permeabilized using 0.2% Triton X-100 in 1X PBS, followed by blocking with 5% Goat Serum-TBS-T (1× TBS with 0.1% Tween-20) to prevent non-specific antibody binding. The cells were then incubated overnight at 4 °C with primary antibodies (Supplementary Table 2). Following this step, Alexa Fluor 488 (green) and 647 (red)-conjugated secondary antibodies (Thermo Fisher, USA) were incubated for 1 hour, and slides were then mounted with coverslips after applying DAPI-containing mounting media (Sigma-Aldrich, USA) to visualize the nuclei. Imaging was performed using a Zeiss Axio Observer 7 microscope or an Olympus Flouview3000 confocal microscope.

### Proximity ligation assay (PLA)

PLA was conducted to investigate direct protein-protein interactions within cells. Approximately 20,000 cells were seeded per well in 8-well chamber slides for this experiment. Following the designated treatments, cells were stained with 50 nM MitoTracker Red CMXRos (Thermo Fisher Scientific, USA) for 15 min under standard culturing conditions. After staining, cells underwent a washing step, and were then fixed with 4% PFA for 15 min at room temperature (RT). Subsequent steps included permeabilization in 0.2% Triton X-100 in 1× PBS for 10 min at RT, followed by PBS washing to remove any residual permeabilization agent. The in-situ PLA experiment was performed per the manufacturer’s guidelines, using the DuoLink kit (Sigma-Aldrich, USA). The primary antibodies used for the PLA are detailed in Supplementary Table 2. After the PLA procedure, coverslips were mounted using DAPI mounting media (Sigma-Aldrich, USA), and imaging was performed using either a Zeiss Axio Observer 7 microscope or an Olympus Flouview3000 confocal microscope.

### MTT cell viability assay

Conditionally expressing WT α-Syn SH-SY5Y cells and NPSCs were seeded in triplicate in a 96-well plate (Corning, NY), with the latter coated with Geltrex. Induction of WT α-Syn expression was carried out for 48 h with Dox. NPSCs were subjected to a 24-h treatment with 10 µM 6OHDA, followed by a media change to either fresh media alone or media containing 10 µM Veliparib, a PARP inhibitor (PARPi) (MEDChemExpress, HY-10129, USA). The MTT assay was then performed following the manufacturer’s instructions provided by Trevigen (Gaithersburg, USA). In brief, 10 μl of MTT reagent was added to each well, and the plates were incubated for 2–4 h, allowing for the formation of visible purple formazan crystals. Subsequently, 100 μL of detergent reagent was added to dissolve the crystals. After an additional 2–4 h of incubation, the absorbance at 570 nm was measured using a microplate reader.

### Mitochondrial membrane potential (MMP) integrity assay

The MMP integrity of conditionally expressing WT α-Syn SH-SY5Y cells was assessed using the TMRE mitochondrial membrane potential assay kit (Abcam ab113852, USA) per the manufacturer’s guidelines. In brief, 20,000 cells were seeded per well in a black clear bottom 96-well plate (Corning 3603, USA). Upon adherence, cells were treated with Dox for 48 h to induce WT α-Syn expression. Subsequently, media containing TMRE was added to the existing media, reaching a final concentration of 750 nM. The cells were incubated with TMRE for 35 min, allowing the dye to accumulate in mitochondria with intact membrane potentials. Following a single wash with 1× PBS, complete DMEM-F12 media without phenol red was added to the cells. The measurement of TMRE fluorescence, indicating mitochondrial membrane potential (MMP) integrity, was conducted at Ex/Em = 549/575 nm using a TECAN Infinite M1000 microplate reader.

### Oxygen consumption rate (OCR) determination using Seahorse

To assess changes in mitochondrial respiration function, the OCR was determined in Dox-inducible WT-α-Syn SH-SY5Y cell lines with and without TOM40 expression supplementation. One cell batch was infected with TOM40 Lv-C-Flag-SV40-eGFP (GeneCopoeia EX-Z2017-Lv203) 72 h before seeding on XF^e^96-well microplates (25,000 cells/well). The XF^e^96 analyzer from Seahorse Bioscience (Agilent Technologies, Waldbronn, Germany) was used to analyze changes in mitochondrial respiration function by performing a Mito Stress Test protocol [43], which is designed to analyze mitochondrial respiration function.

### Mitochondrial long amplification PCR (LA-PCR) based genome integrity analysis

Genomic DNA was extracted from cell lines and tissue samples using the Qiagen Blood and Tissue kit, following the manufacturers protocol. LA-PCR was performed using LongAmp Taq DNA polymerase (NEB) and two sets of primers listed in Supplementary Table 5, each designed to amplify a span of approximately 10 kb within the mitochondrial genome. Primer sets mtLA 5999-1481 and mtLA 179–9231 were employed for cell and tissue samples, respectively, while primer set 7601–16401 was utilized for both types of samples. PCR was performed at 94 °C for 2 min followed by 27 cycles at 94 °C for 30 s, 58 °C (mtLA 5999-1481) or 53 °C (mtLA 7601–16401, 179–9231) for 30 s, and 65 °C for 9 min, and a final elongation step 65 °C for 13 min.

Additionally, a control PCR amplification targeting a shorter 250 bp segment of the human ND1 gene was also conducted. Following the PCR amplifications, the long amplicon products were separated on a 0.8% agarose gel, whereas the short amplicon products were separated on a 2% agarose gel. For independent quantitation of the amplified DNA, Quant-iT PicoGreen (Invitrogen P7589, USA) fluorescence measurements were utilized to enable accurate quantification of double-stranded DNA in solution.

### Mitochondrial DNA NGS sequencing using REPLI-g

The mtDNA genome from WT-α-Syn expressing cells was amplified using REPLI-g Mitochondrial DNA kit (Qiagen, Germantown, MD) according to the manufacturer’s recommendation and the protocol published [44, 45]. The resulting PCR product was sequenced on Illumina HiSeq platform using GENEWIZ next-generation sequencing service. The obtained data were analyzed to identify any changes in the DNA sequence, including point mutations, insertions, deletions, and other genetic variants. The samples were prepared for sequencing by pooling amplified DNA from three controls and three PD patient tissue and the sequencing was independently performed twice.

### Statistical analysis

Each set of data presented in this study is derived from a minimum of three independent experiments were calculated as means ± standard error of the mean (SEM). Statistical analyses were conducted using GraphPad Prism software 9, utilizing both ANOVA and Student’s *t*-tests to discern significant differences.

## Results

### Reduced TOM40 protein levels associated with α-Syn accumulation are independent of transcriptional regulation

The accumulation of α-Syn within mitochondria has been extensively linked to mitochondrial dysfunction including depolarization of the mitochondrial membrane, reduced activity of OXPHOS complexes, and mtDNA damage. Previous studies have also reported reduced TOM40 protein levels, a critical component of the mitochondrial outer membrane translocase complex, in PD brains and transgenic mice expressing α-Syn [27]. However, the functional relationship between α-Syn accumulation and TOM40 reduction has not been explored until now.

To investigate the underlying mechanism behind TOM40 reduction, we assessed TOM40 protein levels in postmortem brain tissue samples from Guam PD, Guam ALS, and Guam control patients obtained from the Binghamton University Biospecimen Archive [34]. Patient demographic details are provided in Supplementary Table 1. To determine whether the protein reduction is specific to TOM40, we examined the TOM20 levels as a control. TOM20, a receptor protein within the TOM complex and plays a similar role in mitochondrial protein import. Importantly, TOM20 protein levels were not affected by α-Syn accumulation, underscoring the specificity of α-Syn-mediated TOM40 reduction [27, 28].

**Fig. 1:**
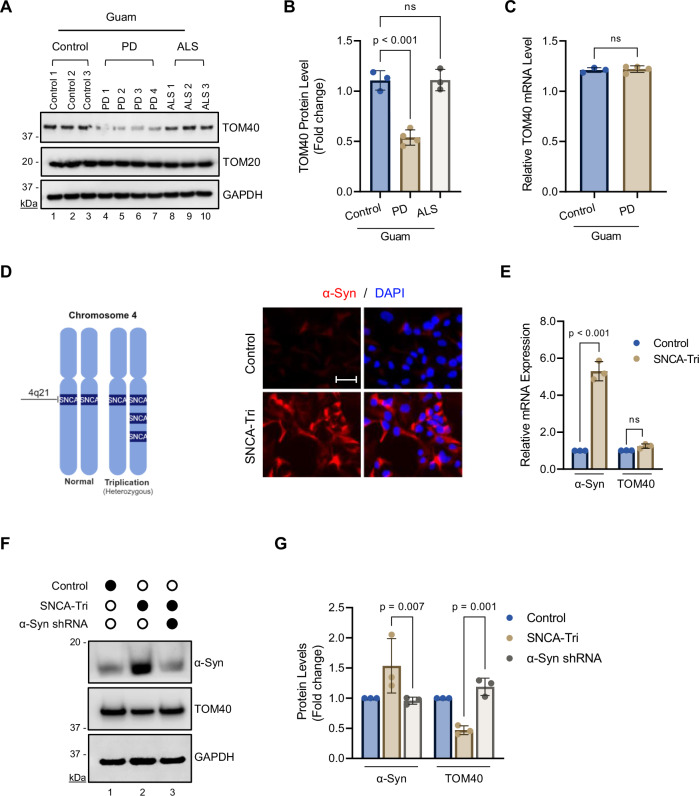
Loss of TOM40 protein levels in PD patient-derived NPSC lines and patient tissue, without corresponding mRNA alterations. **A** Representative immunoblotting of Guam non-neurological controls (*n* = 3) and Guam PD (*n* = *4*) and Guam ALS (*n* = 3) brain tissue extracts. **B** Densitometry analysis demonstrating a reduction in Guam PD (*n* = *4*) TOM40 protein level (Lns 4–7), while TOM20 remains unaffected. **C** RT-qPCR showing consistent TOM40 mRNA levels compared to TOM20 in both Guam non-neurological controls (*n* = 3) and PD brain tissue (*n* = *4*). **D** Schematic diagram of PD patient-derived neural progenitor stem cells (NPSCs) with SNCA triplication (SNCA-Tri), which possess four functional copies of the SNCA gene on chromosome 4 (created with BioRender.com). Representative immunofluorescence confirming elevated α-Syn protein expression (Ab: α-Syn 3H2897, Santa Cruz) in SNCA-Tri NPSCs. Scale bar = 50 µm. **E** RT-qPCR results indicating TOM40 mRNA expression levels in the SNCA-Tri NPSCs cell line. **F**. Representative immunoblot of whole-cell extracts from non-neurological control and SNCA-Tri NPSCs following shRNA-mediated α-Syn knockdown (Ab: α-Syn 204, Biolegend). **G** Densitometric analysis shows reduced TOM40 protein level in SNCA-Tri cells (Ln 2) and significant recovery of TOM40 protein level (Ln 3) upon reduction of α-Syn protein. Data are presented as mean ± s.e.m. from three independent experiments. Statistical analyses were performed using one-way ANOVA (**B**), Student’s *t*-test (**C**), and two-way ANOVA (**E**, **G**). ns non-significant (*p* > 0.05).

Furthermore, immunoblot analysis showed a specific decrease in TOM40, while TOM20 levels remained unchanged in Guam PD brain tissue (Fig. 1A Lns 4–7; Fig. 1B), accompanied by an increase in α-Syn aggregates (Supplementary Fig. 1A Lns 3–4). No reduction in TOM40 protein level was observed in Guam non-neurological controls or ALS samples (Fig. 1A Lns 1–3, 8–10; Fig. 1B). These findings suggest that the observed reduction in TOM40 is specific to the Guam PD pathology.

To test the possibility of transcriptional regulation behind TOM40 reduction, we measured TOM40 and TOM20 mRNA levels in Guam PD postmortem brain tissues by qRT-PCR (Fig. 1C). The results showed that TOM40 mRNA level normalized to TOM20 mRNA level did not differ, indicating that TOM40 degradation occurs post-translationally. To investigate this further, we utilized a PD patient-derived SNCA-Tri line containing four functional *SNCA* gene copies, three mutant alleles, and one wild-type allele (Fig. 1D). Notably, despite having a sixfold higher α-Syn mRNA level, the SNCA-Tri line exhibited stable TOM40 mRNA levels compared to the control (Fig. 1E), but reduced TOM40 protein level compared to the control line (Fig. 1F Ln 2; Fig. 1G). Additionally, downregulating α-Syn in the SNCA-Tri line resulted in increased TOM40 protein levels (Fig. 1F Ln 3; Fig. 1G) but did not lead to any changes in the mRNA levels (Supplementary Fig. 1D).

### Factors inducing α-Syn oligomerization exacerbate TOM40 loss

**Fig. 2:**
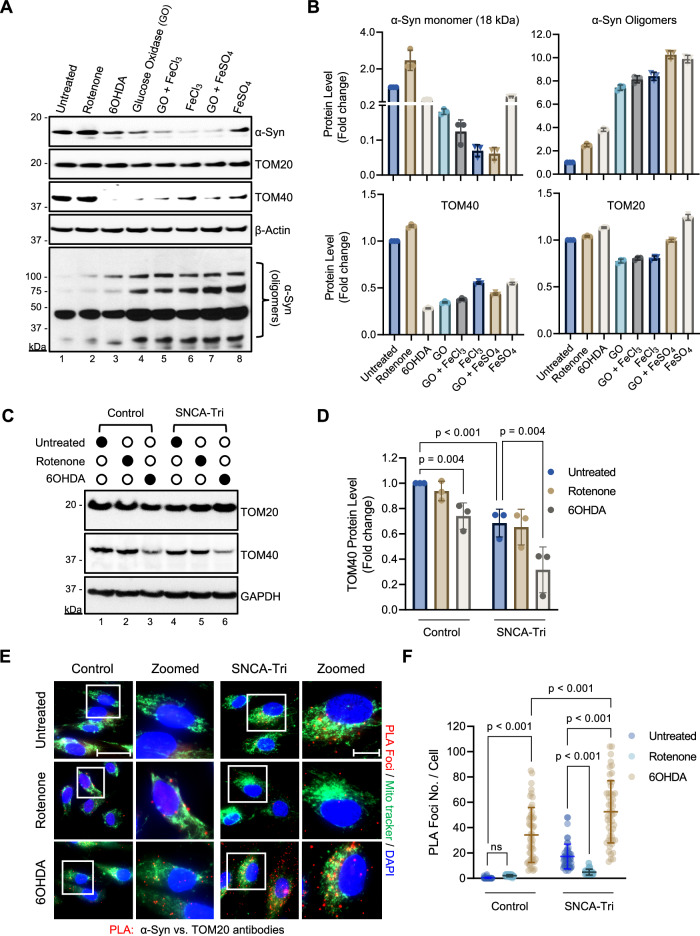
α-Syn aggregation-promoting factors affect TOM40 but not TOM20 protein levels. **A** Representative immunoblotting of whole-cell extracts from SH-SY5Y cells overexpressing α-Syn after exposure to PD-linked etiological agents (10 µM rotenone, 10 µM 6OHDA, 50 µM glucose oxidase (GO), 50 µM FeCl_3_, and 50 µM FeSO_4_). **B** Densitometry analysis demonstrating protein levels for 18 kDa α-Syn monomers (Ab: α-Syn EP1646, Millipore), α-Syn oligomers α-Syn (Ab: α-Syn EP1646, Millipore), TOM40, and TOM20. **C** Representative immunoblotting of whole-cell extract from non-neurological Control and SNCA-Tri patient-derived NPSCs treated with either 10 µM rotenone or 6OHDA. **D** Densitometry analysis revealed lower TOM40 protein levels in control and SNCA-Tri NPSCs following 10 µM 6OHDA exposure (Lns 3, 6). **E** Proximity Ligation Assay (PLA) images highlighting the interaction between α-Syn (Ab: α-Syn 3H2897, Santa Cruz), and TOM20 (red foci), co-stained with Mito tracker (green). Scale bar = 10 µm; scale bar zoomed images = 5 µm. **F** PLA analysis histogram indicates a significant difference in PLA foci counts between control and SNCA-Tri NPSCs following 6OHDA and rotenone exposure. Data are presented as mean ± s.e.m. from three independent experiments; quantification of PLA foci was derived from 50 cells. All statistical analyses were performed using two-way ANOVA. ns = non-significant (*p* > 0.05).

In normal cells, α-Syn predominantly resides in the cytoplasm but redistributes to the mitochondria under oxidative stress, with its impact on outer mitochondrial membrane (OMM) proteins yet to be explored [2, 46, 47]. To investigate the impact of α-Syn accumulation under oxidative stress on OMM proteins, we evaluated the effects of PD-associated neurotoxins and pro-oxidant metals—known for generating mitochondrial and cytosolic ROS and fostering α-Syn aggregation in PD models—on the levels of TOM40 and TOM20 (Fig. 2A) [11, 48, 49]. Immunoblot analysis revealed a significant decrease in TOM40 levels in SH-SY5Y cells overexpressing ectopic α-Syn and exposed to 6OHDA (Fig. 2A Ln 3; Fig. 2B), GO (Fig. 2A Ln 4; Fig. 2B), FeCl_3_ (Fig. 2A Ln 6; Fig. 2B), or FeSO_4_ (Fig. 2A Ln 8; Fig. 2B) treatments, while TOM20 levels remained unaltered. Interestingly, the reduction in TOM40 levels correlated strongly with decreased α-Syn monomer levels and an increase in α-Syn oligomer formation (Fig. 2A Lns 3–8; Fig. 2B), as indicated by high-mobility bands in the immunoblots probed with the α-Syn antibody.

Prompted by reports indicating reduced viability and stress resistance in comparable cell lines exposed to toxicants such as rotenone and 6OHDA, we conducted a thorough examination to assess the impact on TOM40 levels in PD patient-derived NPSCs harboring SNCA gene triplication [50, 51]. The reduction in TOM40 protein levels observed in 6OHDA-treated NPSCs, particularly in the SNCA-Tri line (Fig. 2C Lns 3, 6; Fig. 2D), mirrored the observations in SH-SY5Y cells overexpressing α-Syn. Importantly, exposure to rotenone did not affect TOM40 levels in these cell models (Fig. 2B Lns 2, 5; Fig. 2D), suggesting that TOM40 degradation is more susceptible to cytoplasmic ROS inducers than mitochondrial ones. Furthermore, Mitotracker-PLA immunofluorescence studies indicated an enhanced interaction between α-Syn and TOM20 (Fig. 2E, F; Supplementary Fig. 2), consistent with prior findings of α-Syn’s mitochondrial translocation under oxidative stress [46]. Additionally, immunoprecipitation experiments (Supplementary Fig. 3) using recombinant α-Syn and mitochondrial lysates from non-neurological control NPSCs revealed a direct interaction with TOM40 and TOM20, but not TOM22, reinforcing the specificity of α-Syn’s selective binding to certain components of the TOM complex. This finding is consistent with previous studies that suggest α-Syn’s specific interaction with TOM20 but not TOM22 within the TOM complex [28]. Altogether, our results provide new insights into how oxidative stress, coupled with α-Syn accumulation, impacts TOM protein activities and levels in PD.

These findings also led us to investigate the mechanisms underlying synthesis-induced TOM40 dysfunction and to develop strategies to counteract the perturbations in TOM40 as potential therapeutic avenues for PD.

### Mitochondrial localization of α-Syn and its influence on TOM40 levels

**Fig. 3:**
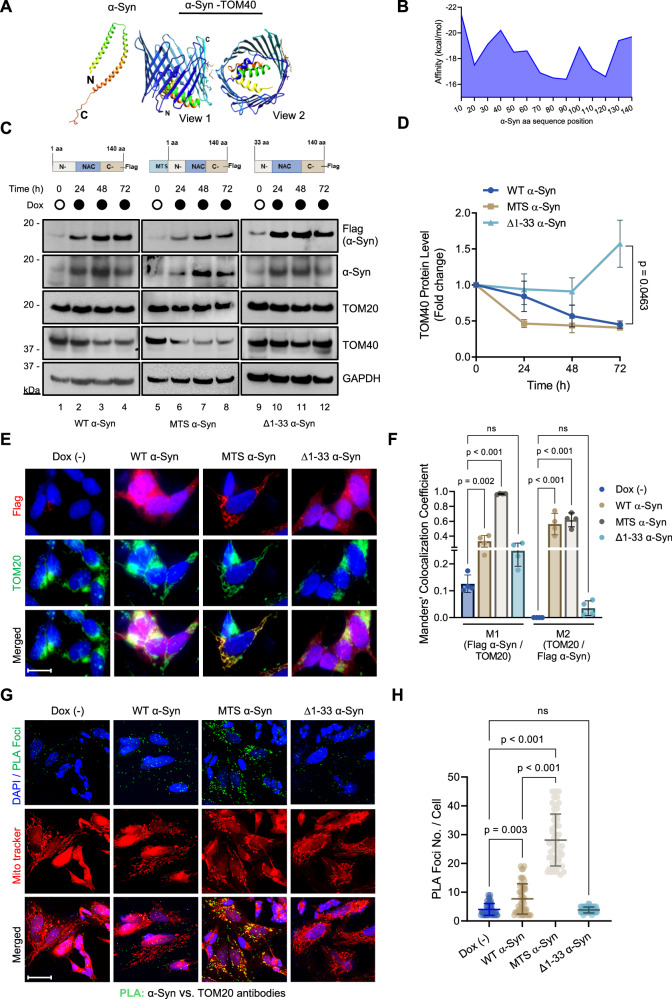
α-Syn’s N-terminus is required for mitochondrial localization and TOM40/TOM20 interaction. **A** α-Syn structure PDB ID:1xq8 divided into 20 residue fragments, docked against TOM40 structure PDB ID: 7CK6 using AutoDockCrankPep. Image created with PyMOL. **B** Graph determining α-Syn residues with a higher binding affinity for TOM40. **C** Schematic representation of vectors used for generating wild-type (WT), mitochondria-targeted signal (MTS), and truncated (Δ1–33) overexpressing α-Syn SH-SY5Y stable cell lines (N N-terminal, NAC nonamyloidogenic component, C C-terminal). Representative immunoblot from WT, MTS, and Δ1-33 α-Syn whole-cell extracts (Ab: α-Syn 204, Biolegend). **D** Densitometry analysis indicates TOM40 protein level are specifically affected in WT (Lns 3,4) and MTS α-Syn (Lns 6–8) overexpressing models. **E** Immunofluorescence images confirming cellular localization of Flag α-Syn (Ab: Anti-DDDDK tag, Abcam) after 48 h Dox induction in WT α-Syn, MTS α-Syn, and Δ1-33 α-Syn. Scale bar = 10 µm. **F** Quantification of Manders’ colocalization coefficient M1 (Fraction of red channel = Flag α-Syn in colocalization with green channel = TOM20) or M2 (Fraction of green channel = TOM20 in colocalization with red channel = Flag α-Syn). **G** PLA images highlighting the interaction between α-Syn (Ab: α-Syn 3H2897, Santa Cruz), and TOM20 in mitochondria (green foci), co-stained with MitoTracker (red). Scale bar = 10 µm. **H** PLA analysis showing increased WT and MTS α-Syn interaction with TOM20. Scale bar = 10 µm. Data are presented as mean ± s.e.m. from three independent experiments; quantification of PLA foci (**H**) was derived from 50 cells. The statistical analyses used Student’s *t*-test (**D**), one-way ANOVA (**F**), and two-way ANOVA (**H**). ns non-significant (*p* > 0.05).

While α-Syn does lack a conventional mitochondrial targeting signal (MTS), its presence in different mitochondrial compartments raises questions about its import mechanism into the mitochondria. Previous studies have shown that α-Syn’s N-terminal 32 amino acids are essential for its mitochondrial translocation, a process that is likely mediated through interaction with TOM40 [2]. To identify the residues contributing to this interaction, we conducted molecular docking studies using the AutoDock CrankPep protein-peptide docking method [52]. Since the program reliably docks peptides raging in lengths from 16 to 20 amino acids, we divided α-Syn’s 140 amino acid sequence into 20 amino acid fragments using the micelle-bound human α-Syn (PDB ID: 1XQ8) as a reference structure (Fig. 3A). The docking results using the TOM40 structure (PDB ID: 7CK6) as the receptor revealed that α-Syn fragment (21–40) exhibited the highest predicted binding affinity for TOM40 residues (Fig. 3B). Notably, α-Syn C-terminal residues demonstrate a lower binding affinity. Overall, the docking results suggest that α-Syn N-terminal residues are crucial for interacting with TOM40.

Building on the docking analysis and existing literature, we designed two inducible expression vectors: one containing a mitochondrial targeting signal (pCW MTS-α-Syn-Flag) and another with a 33 amino acid deletion of α-Syn’s N-terminal region (pCW Δ1-33-α-Syn-Flag) (Fig. 3C). These vectors were used to establish stable SH-SY5Y cell lines as a proof-of-concept model to test whether inhibition of α-Syn translocation into the mitochondria affects TOM40 levels or if targeted mitochondrial accumulation of α-Syn exacerbates TOM40 loss. Utilizing these inducible SH-SY5Y lines enables post-differentiation induction of α-Syn expression, preventing non-physiological α-Syn levels during the differentiation process, known to impact neuronal differentiation, resulting in poor neuronal morphology and shorter neurites [53]. Western blot analysis of Δ1-33 α-Syn SH-SY5Y cells showed no significant alteration in TOM40 protein levels (Fig. 3C Lns 10-12; Fig. 3D). On the other hand, targeting the expression of α-Syn into the mitochondria led to an enhanced reduction in TOM40 levels after 24 h of induction (Fig. 3C Lns 6–8, 3D), while TOM20 protein levels exhibited no alterations despite the enforced expression of α-Syn in the mitochondria. No significant changes in TOM40 mRNA levels were observed at any tested time points during α-Syn induced expression (Supplementary Fig. 3A).

Investigating the impact of oligomeric forms of α-Syn, especially small soluble α-Syn aggregates, is crucial for understanding key pathological aspects of PD development [54]. Using the anti-oligomer A11 polyclonal antibody, we detected the accumulation of α-Syn oligomers (>25 kDa) in a dose-dependent manner upon induction of α-Syn expression, with MTS-α-Syn expressing cells showing a more pronounced accumulation (Supplementary Fig. 3B Lns 6–8). In contrast, cells expressing Δ1-33 α-Syn for a longer period (120 h) form a distinct oligomerization pattern compared to the wild-type or MTS version (Supplementary Fig. 3C). These results align with the reduced TOM40 levels observed in (Fig. 3C Lns 6–8), suggesting a potential association between α-Syn oligomeric forms and TOM40 loss. Immunofluorescence images and Manders’ colocalization coefficient analysis (Fig. 3E, F) confirmed the mitochondrial accumulation of ectopic MTS-α-Syn. Additionally, PLA analysis revealed an increased interaction between WT and MTS-α-Syn with TOM20 (Fig. 3G, H) and WT α-Syn with TOM40, observed at 6 and 12 h following Dox induction (Supplementary Fig. 3D).

Overall, these findings suggest that TOM40 loss in the context of Guam PD pathology is influenced by two key factors: α-Syn’s interaction with OMM proteins via its N-terminal residues, and accumulation of α-Syn oligomers, particularly at the mitochondria.

### Proteasomal degradation as a key mechanism in α-Syn-induced TOM40 loss

**Fig. 4:**
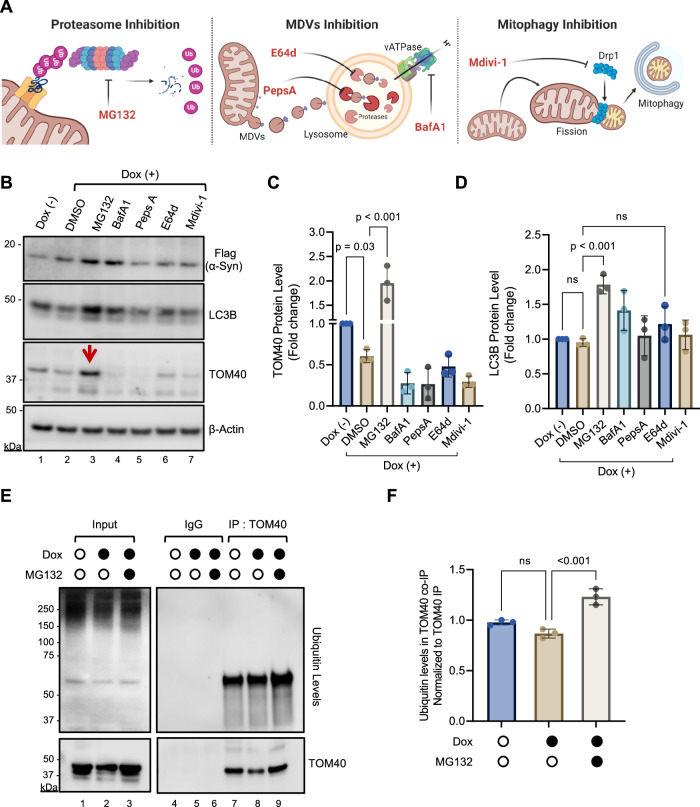
Involvement of the ubiquitin-proteasome pathway in α-Syn-induced TOM40 loss. **A** Schematic diagram depicting the different inhibitors used to test potential mitochondrial quality control pathways involved in TOM40 loss. Inhibitors: MG132, Bafilomycin A1 (BafA1), Pespstatin (PepsA), E64d, Mdivi. Scheme created with BioRender.com. **B** Representative immunoblot of WT α-Syn cells pre-treated for 24 h with different inhibitors followed by 48 h of Dox-induced α-Syn expression. **C** Densitometry analysis showing high TOM40 level (Ln 3, red arrow) in MG132 treated WT α-Syn cell. **D** Densitometry analysis shows high LC3B level in MG132 (Ln 3) and bafilomycin A1 (Ln 4) treated WT α-Syn cells. **E** Representative immunoblot of endogenous TOM40 co-IP with ubiquitin from WT α-Syn cells pre-treated with MG132 for 24 h and induced with Dox for 48 h. **F** Densitometry analysis of ubiquitinylated protein levels in TOM40 co-IP. Data are presented as mean ± s.e.m. from three independent experiments. Statistical analysis was performed using one-way ANOVA.

In our investigation into the mechanisms underlying TOM40 loss in response to α-Syn accumulation, we explored three pathways (Fig. 4A) associated with the selective degradation of OMM proteins: the ubiquitin-proteasome system (UPS), mitochondrial-derived vesicles (MDVs), and mitophagy [42, 55, 56]. Our findings revealed a significant stabilization of TOM40 protein levels in the presence of MG132 (Fig. 4B red arrow Ln 3; Fig. 4C). We also observed increased interaction between α-Syn and TOM40 (Supplementary Fig. 4A), suggesting that proteasomal degradation is a primary mechanism regulating TOM40 levels in the context of α-Syn accumulation. Notably, the stabilization of TOM40 was unique to proteasome inhibition, as other inhibitors failed to produce the same effect, highlighting the specificity of the UPS in this process.

We also assessed the effectiveness of these inhibitors by observing a notable increase in microtubule-associated protein 1 A/1B-light chain 3B (LC3B). Interestingly, LC3B levels were lower in α-Syn-expressing cells compared to the Dox (-) control (Fig. 4B Ln 2; Fig. 4D). This reduction aligns with previous reports of decreased LC3B levels in α-Syn-overexpressing mid-brain neurons and PD patients’ cerebrospinal fluid, suggesting that α-Syn can reduce LC3B levels by reducing its synthesis rather than increasing its degradation by lysosomes [57, 58].

However, when inhibitors were applied, all of them led to elevated LC3B levels compared to vehicle-treated α-Syn-expressing cells, with the strongest effects observed after MG132 and BafA1 treatments (Fig. 4B Lns 3, 4; Fig. 4D). Although increased LC3B is typically associated with mitophagy, recent evidence suggests that MDVs might serve as an alternative or compensatory mechanism for mitochondrial quality control when mitophagy is compromised [59].

Interestingly, BafA1, a vacuolar ATPase inhibitor that specifically disrupts lysosomal acidification and causes the cytosolic accumulation of MDVs [41, 60], does not inhibit TOM40 degradation or prevent α-Syn accumulation. In contrast, E64d, a lysosomal protease inhibitor also known to promote MDVs accumulation in the cytosol [42], partially restored TOM40 protein levels compared to the other lysosomal inhibitors tested. Nonetheless, TOM40 levels in E64d-treated cells were similar to those observed in DMSO-treated Dox (+) cells, indicating limited recovery.

Further evidence supporting UPS involvement was obtained by immunoblotting TOM40 immunoprecipitates with a ubiquitin antibody, revealing a ubiquitinated protein band at the TOM40 size (Fig. 4E Lns 7–9; Fig. 4F). Although there were no significant differences in ubiquitinated protein levels between Dox (−) and Dox (+) conditions, likely due to the overall reduction in TOM40 caused by α-Syn accumulation, proteasome inhibition with MG132 increased ubiquitinated proteins. These data suggest that TOM40 is ubiquitinated and that this ubiquitination is markedly increased in cells treated with proteasome inhibitor. The results also indicate that TOM40 undergoes proteasomal degradation via ubiquitination, and the elevated ubiquitination levels in the presence of MG132 underscore the role of the proteasome in TOM40 turnover.

In summary, our findings demonstrate that proteasomal degradation is a key driver of TOM40 loss in the context of α-Syn accumulation. Additionally, these results underscore the complex nature of mitochondrial quality control in neurodegenerative diseases, where multiple pathways, including the UPS, MDVs, and mitophagy, work together to clear damaged mitochondria.

### Impact of α-Syn pathology on mitochondrial genome integrity

**Fig. 5:**
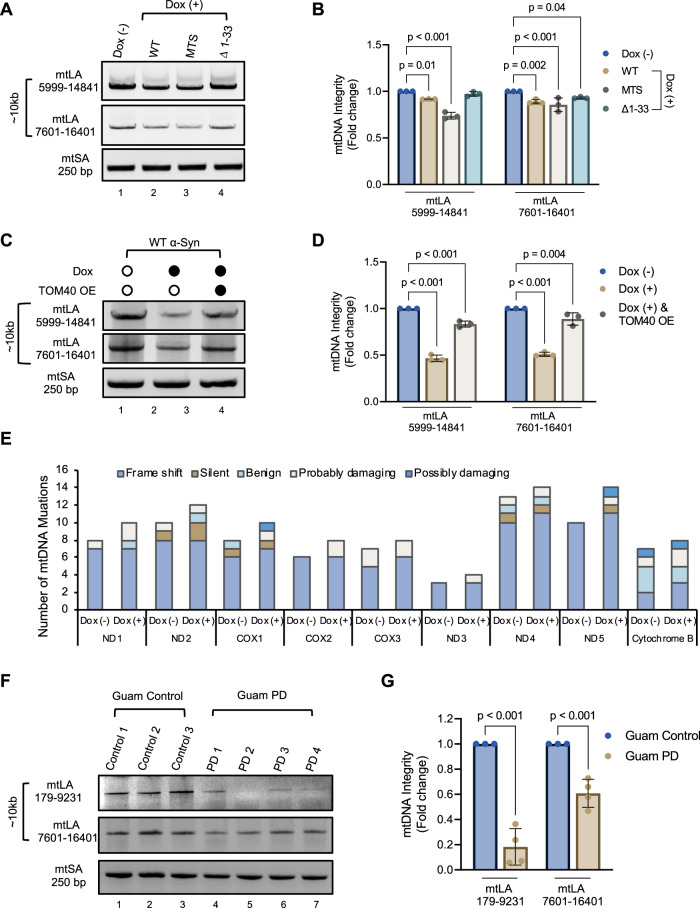
Impact of α-Syn accumulation on mitochondrial DNA (mtDNA) integrity. **A** Electrophoresis of long amplification PCR (LA-PCR) products on a 1% agarose gel, showing both long ~10 Kb (mtLA) and short ~250 bp (mtSA) mtDNA amplicons for WT, MTS, and Δ1–33 overexpressing α-Syn SH-SY5Y stable cell lines. **B** Quantification of mtDNA integrity using PicoGreen fluorescence, presented as fold change relative to Dox (−) cells. **C** LA-PCR 1% agarose gel for WT α-Syn SH-SY5Y stable cell line with TOM40 overexpression (TOM40 OE). **D** Quantification of mtDNA integrity using PicoGreen fluorescence, presented as fold change relative to Dox (−) cells. **E** mtDNA sequencing analysis: mutation frequency graph summarizing unique mutations in protein-coding genes by type and severity. Frameshifts result from insertions or deletions, while silent mutations code for the same amino acid. Severity is classified as benign, probably damaging, or possibly damaging by PolyPhen-2 analysis. **F** LA-PCR of brain tissue DNA from Guam PD patients on a 1% agarose gel, showing ~10 Kb mtLA and ~250 bp mtSA mtDNA amplicons to evaluate mtDNA integrity. **G** Quantification of mtDNA integrity via densitometry analysis is presented as fold change tissue (Guam non-neurological controls, *n* = *3;* Guam PD, *n* = *4*). Data are presented as mean ± s.e.m. from three independent experiments. Statistical analyses were performed using two-way ANOVA (**B**, **D**, **G**) and Student’s *t*-test (**H**).

To assess the impact of α-Syn pathology on mitochondrial genome integrity, we used long amplification (LA-PCR) analysis. Separation of PCR amplicons in 1% agarose gel electrophoresis (Fig. 5A) revealed a significant reduction in mtDNA amplification in cells overexpressing WT α-Syn (Fig. 5A Ln 2) and MTS α-Syn (Fig. 5A, Ln 3) compared to control (Fig. 5A Ln 1) or Δ1-33 α-Syn (Fig. 5A Ln 4) cells. Quantitation of PCR products using an independent, highly sensitive PicoGreen based plate reader method confirmed a marked reduction in mtDNA integrity in WT and MTS α-Syn overexpressing cells (Fig. 5B). The reduction in LA-PCR products is indicative of PCR DNA polymerase blocking lesions in DNA, predominantly involving DNA strand breaks, as well as DNA crosslinks and bulky base damages. Therefore, reduction in mtDNA amplification suggests that residues located in the N-terminal region not only facilitate α-Syn’s translocation into the mitochondria but also contribute to its adverse effects on mtDNA integrity. Consequently, averting the buildup of mitochondrial α-Syn by N-terminal deletion not only safeguards TOM40 levels but also preserves the integrity of mtDNA.

We further assessed the impact of restoring TOM40 levels in WT α-Syn cells (Fig. 5C, D). Our results show that supplementing TOM40 indeed restores mitochondrial genome integrity, further supporting the connection between α-Syn-mediated TOM40 loss and mitochondrial genome damage.

To gain deeper insights into the potential link between mtDNA instability and α-Syn accumulation, we conducted mitochondrial DNA sequencing to quantify insertions, deletions, and mutations in WT α-Syn cells. Our analysis identified several unique mutations in the ND2, COX1, ND4, and ND5 genes. The severity assessment using the PolyPhen-2 online tool revealed the impact of these mutations (Fig. 5E). A comparison between Dox (−) and Dox (+) WT α-Syn cells revealed a total of 148 mutations with a rate of 8.9 mutations per 1 kb in Dox (−), while 167 mutations were present in Dox (+) with a mutation rate of 10 mutations per 1 kb. Specifically, 26 unique mutations were reported in Dox (+) WT α-Syn cells mitochondria at a rate of 1.5 mutations per 1 kb. Analyzing the distribution of mutations in the coding region of the mitochondrial genome in Dox (+) WT α-Syn cells, we found varied rates across different genes: ND1 (2.1/kb), ND2 (1.9/kb), COX1 (1.9/kb), COX3 (1.2/kb), ND3 (2.8/kb), ND4 (0.72/kb), ND5 (2.1/kb), ND6 (3.9/kb), and Cytb (0.8/kb).

Furthermore, we assessed mtDNA integrity in brain tissue samples from Guam PD patients exhibiting α-Syn pathology and compared them to three non-neurological controls using LA-PCR (Fig. 5F). Agarose gel electrophoresis densitometry analysis (Fig. 5G) revealed a significant increase in mtDNA damage within the Guam PD patient samples (Fig. 5F Lns 4–7), contrasting with Guam non-neurological controls (Fig. 5F Lns 1–3). Altogether these findings emphasize a distinct correlation between mtDNA instability and α-Syn proteinopathy.

### TOM40 supplementation partially counters α-Syn-induced defects in mitochondrial bioenergetics

**Fig. 6:**
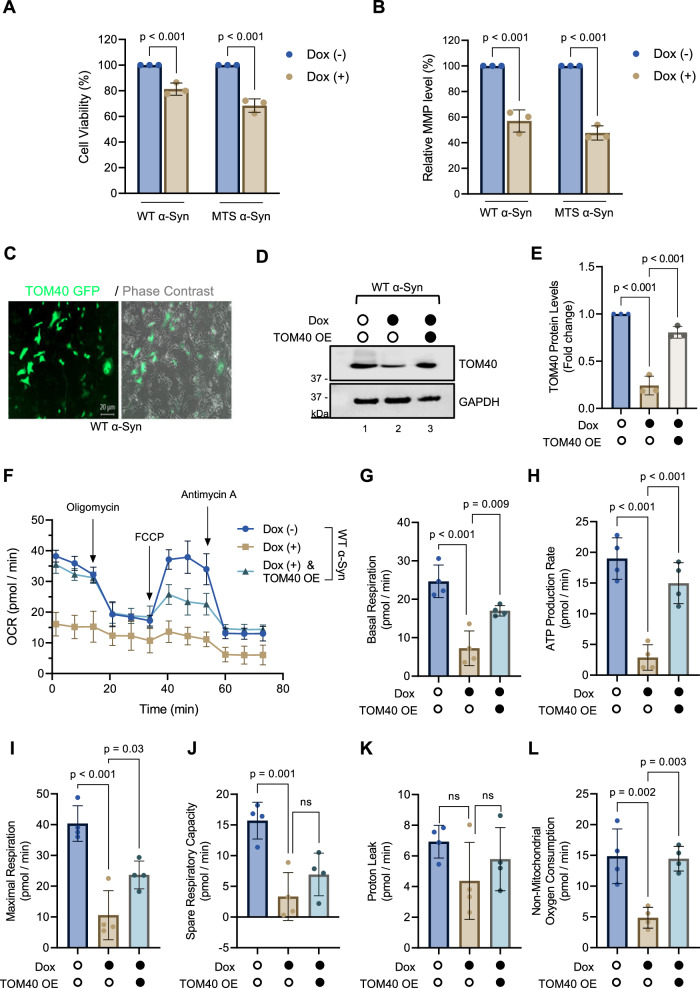
TOM40 supplementation partially mitigates α-Syn-induced mitochondrial defects. **A** Assessment of cell viability with MTT assay after 48 h of induced α-Syn overexpression. **B** Measurement of mitochondrial membrane potential with TMRE assay results following 48 h of induced α-Syn overexpression. **C** Representative epifluorescence/phase-contrast image of WT α-Syn cells infected with TOM40 eGFP Lv-C-Flag-SV40-eGFP infection, scale bar = 20 µm. **D**, **E** Representative immunoblot and densitometry analysis demonstrating TOM40 overexpression level post-infection. **F** Seahorse analysis presenting an overview of Oxygen Consumption Rate (OCR) in WT α-Syn Dox (-), Dox (+), and Dox (+) plus TOM40 overexpression (TOM40 OE) cells during the mitochondrial respiration test. **G** Basal respiration in WT α-Syn overexpressing cells. **H** ATP-linked respiration. **I** Maximal respiration was assessed following mitochondrial uncoupling by FCCP. **J** Spare respiratory capacity is determined by subtracting basal respiration from maximal respiration in WT α-Syn overexpressing cells. **K** Proton leakage was evaluated after inhibiting complex III via antimycin-A. **L** Non-mitochondrial oxygen consumption. Data (**A**, **B**, **E**) are presented as mean ± s.e.m. from three independent experiments, and data (**F**–**L**) are presented as presented mean ± s.e.m. from four independent experiments. The statistical analyses were performed using two-way ANOVA (**A**, **B**) and one-way ANOVA (**E**–**L**). non-significant (*p* > 0.05).

Considering the impact of α-Syn on cellular health, particularly focusing on cell viability (Fig. 6A) and mitochondrial membrane potential (Fig. 6B), we aimed to assess the functional consequences of overexpressing WT α-Syn in the absence or presence of ectopic TOM40 supplementation (Fig. 6C–E). Utilizing the Seahorse XFe96 analyzer, we conducted a real-time assessment of mitochondrial respiratory function, quantifying oxygen consumption rates (OCR) to measure the mitochondrial bioenergetic efficiency.

Our findings reveal that WT α-Syn overexpression in SH-SY5Y cells, without concurrent TOM40 supplementation, significantly diminishes OCR alongside all tested parameters of respiratory function (Fig. 6F). This reduction highlights a clear compromise in mitochondrial efficiency attributable to α-Syn overexpression. In contrast, cells overexpressing WT α-Syn and supplemented with TOM40 expression exhibited a substantial enhancement in OCR (Fig. 6F), indicating an improvement in mitochondrial bioenergetics. This improvement extended across several respiratory parameters, including basal respiration (Fig. 6G), ATP production (Fig. 6H), and maximal respiration (Fig. 6I), where we observed significant improvements. Notably, non-mitochondrial oxygen consumption also showed marked enhancement (Fig. 6L).

However, the spare respiratory capacity, which acts as a buffer during increased energy demand, remained adversely affected in cells with elevated α-Syn levels, indicating that TOM40 supplementation, while beneficial, could not completely mitigate this specific deficit (Fig. 6J). Furthermore, our results showed that proton leak rates were not significantly altered by either α-Syn overexpression or TOM40 supplementation (Fig. 6K), suggesting that some aspects of mitochondrial function remain unaffected by these modifications.

Collectively, these outcomes underscore that while TOM40 supplementation does not completely reverse the mitochondrial impairments induced by α-Syn overexpression, it does significantly ameliorate several key aspects of mitochondrial bioenergetics. This partial but meaningful recovery suggests a promising avenue for addressing mitochondrial deficits linked to α-Syn pathology, while also suggesting a need for additional strategies to mitigate such deficits.

### PARP inhibition as a strategy to restore TOM40 levels and mitigate α-Syn toxicity

**Fig. 7:**
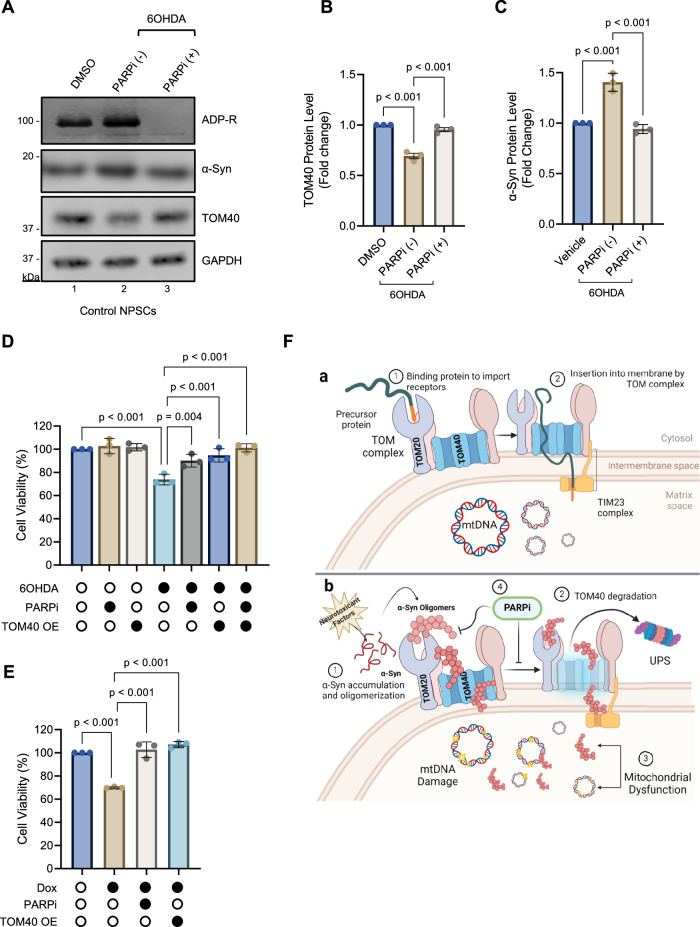
Restoration of TOM40 protein levels by ADP-Ribosylation inhibitors (PARPi) in 6OHDA-induced cellular stress and α-Syn expression. **A** Representative immunoblot of whole-cell extract from control NPSCs exposed to 10 µM 6OHDa for 24 h and subsequently treated with 10 µM Veliparib (PARPi) for an additional 24 h. **B**, **C** Densitometry analysis revealed a significant recovery in TOM40 protein level and reduced α-Syn protein level (Ab: α-Syn 204, Biolegend) following PARPi treatment (Ln 3). **D** Impact of PARPi and TOM40 overexpression on cell viability in SH-SY5Y cells under 6OHDA-induced toxicity. **E** Effect of PARPi and TOM40 overexpression on cell viability in WT α-Syn cells induced with dox for 48 h. **F** Schematic diagram illustrating mechanisms of TOM40 loss induced by α-Syn pathogenesis: (**a**) Normal protein import into mitochondria under physiological conditions. (**b**) In the presence of genetic mutations or neurotoxicants, α-Syn accumulates and forms oligomers (1), triggering TOM40 degradation via the UPS pathway (2), leading resulting in mitochondrial dysfunction (3). Inhibition of PARP1 serves to restore TOM40 levels, representing a promising strategy to counteract mitochondrial dysfunction and prevent cell death triggered by ROS toxicity and pathological α-Syn accumulation (4). Data (**B**–**E**) are presented as mean ± s.e.m. from three independent experiments and were analyzed by one-way ANOVA.

Building upon recent findings that suggest that PARP inhibition promotes the degradation of α-Syn aggregates via the autophagy-lysosomal pathway in PD models [33], here, we explored the potential of PARP inhibition in countering α-Syn-mediated TOM40 protein loss resulting from exposure to 6OHDA and elevated α-Syn expression. We treated control NPSCs with 6OHDA and then evaluated the effect of Veliparib, a potent PARP inhibitor (PARPi) known for its ability to cross the blood-brain barrier [61], on TOM40 levels. Veliparib effectively inhibited PARP activity, as demonstrated by a reduction in Poly/Mono-ADP ribosylated (ADP-R) proteins. The efficacy of Veliparib was further supported by a significant restoration of TOM40 protein levels compared to untreated cells (Fig. 7A Ln 2 vs 3, 7C) concomitant with decreased 18 kDa α-Syn protein levels (Fig. 7C). These findings not only validate Veliparib’s expected inhibition of PARP, but also suggest a mechanism by which PARP inhibition contributes to TOM40 stabilization by reducing α-Syn protein levels.

Finally, we compared the effectiveness of TOM40 supplementation and PARPi treatment on the viability of two PD in vitro models: the 6OHDA-induced (Fig. 7D) and α-Syn overexpression model (Fig. 7E). Interestingly, both TOM40 supplementation and PARPi treatment demonstrated similar efficacy in enhancing cell viability. This finding is particularly significant as it highlights the potential of two independent therapeutic pathways for mitigating the deleterious effects of α-Syn-induced mitochondrial dysfunction and subsequent cell death (schematically illustrated in Fig. 7Fa, b).

## Discussion and conclusion

This study elucidates the mechanisms that contribute to the reduction of the TOM40 protein on the OMM, which is linked to α-Syn pathology. Furthermore, this study highlights the implications of TOM40 loss for mitochondrial dysfunction in Guam PD. Our findings demonstrate that α-Syn accumulation uniquely influences TOM40 protein levels, a process independent of transcriptional regulation. While analysis of Guam PD patient brain tissue samples and age-matched control samples aligns with earlier research, confirming that TOM40, not TOM20, is affected in cases with higher α-Syn levels [27], this is the first report of such depletion of TOM40 in Guam PD patient brain tissues. Furthermore, a comparative analysis of Guam PD with Guam ALS, without α-Syn proteinopathy revealed that loss of TOM40 is only observed in Guam PD but not in Guam ALS, underscoring its susceptibility to α-Syn-induced alterations. Additionally, our observations from various PD cell models emphasize TOM40 mRNA stability irrespective of α-Syn mRNA levels, which is consistent with patient brain tissue results. This underscores TOM40’s susceptibility to alterations in protein levels, distinguishing it from other mitochondrial proteins such as TOM20.

Another crucial observation is the increased reduction of TOM40 in the presence of PD-associated neurotoxicants and ROS inducers. Our findings demonstrate an increased TOM40 loss when α-Syn expression is coupled with ROS-generating agents such as 6OHDA, which also exhibit reduced levels of monomeric α-Syn and increased oligomer formation. Neurotoxic agents such as rotenone and 6OHDA generate intracellular ROS, with rotenone primarily inducing mitochondrial ROS through direct COXI inhibition, while 6OHDA undergoes rapid autoxidation in the cytosol, leading to hydrogen peroxide accumulation in this compartment [6, 62]. Therefore, our results from PD cell models underscore the susceptibility of TOM40 to cytoplasmic ROS inducers, providing valuable insights into the impact of oxidative stress on TOM protein activities.

Additionally, the interaction between α-Syn and TOM20 significantly increased upon 6OHDA treatment, while the lack of interaction with TOM22 suggests that α-Syn selectively associates with specific outer mitochondrial membrane proteins. This finding aligns with recent evidence showing that pathogenic α-Syn aggregates preferentially bind to mitochondria through increased interaction with TOM20 [63].

Importantly, TOM40 degradation is exacerbated by mitochondrial-localized α-Syn, as evidenced by the preservation of TOM40 protein levels in cells expressing Δ1-33-α-Syn, which inhibits the translocation of α-Syn into mitochondria. In contrast, TOM20 protein levels were not influenced by either the targeting of α-Syn into the mitochondria or the deletion of the first 33 amino acids containing the potential intrinsic MTS of α-Syn. The absence of the first 33 amino acids in α-Syn emphasizes not only their significance for the mitochondrial accumulation of α-Syn but also for the interaction between α-Syn and TOM complex proteins. Moreover, the simultaneous occurrence of intracellular α-Syn oligomeric species formation alongside TOM40 loss supports the notion that pathogenic forms of α-Syn instigate significant disturbances in the physical structure and integrity of the OMM. These changes may impact the OMM’s ability to regulate molecule transport, maintain an electrochemical gradient, and participate in vital cellular processes. This implication aligns with earlier studies that demonstrated alterations in the inner mitochondrial membrane resulting from the accumulation of α-Syn within the mitochondria [4]. Therefore, the accumulation of α-Syn oligomers, particularly in the mitochondria, is associated with TOM40 loss, consistent with their well-established pathological role in PD.

Our investigation also implicates the UPS in α-Syn-mediated TOM40 degradation, likely through the α-Syn-triggered ubiquitination of TOM40. The involvement of the UPS pathway in α-Syn-mediated TOM40 loss suggests an adaptive response to prevent organelle-wide damage, a notion supported by evidence highlighting the role of mitochondria-associated degradation (MAD) pathway, which selectively removes damaged OMM proteins rather than eliminating entire organelles through mitophagy processes [64, 65]. Moreover, based on our observations, one possible explanation for the selective degradation of TOM40, but not TOM20, may involve its critical role in mitochondrial quality control mechanisms. When mitochondria are damaged, the accumulation of PINK1 at the outer mitochondrial membrane is a key signal that activates the mitophagy pathway [66], where TOM40 assists in this process [67]. If α-Syn disrupts this mechanism [68], it could lead to TOM40’s degradation through the UPS, as our study suggests. Conversely, TOM20 may be less susceptible to degradation, as it may not play as direct a role in these quality control mechanisms. Additionally, previous studies have shown that overexpression of TOM20 can have protective effects against α-Syn-induced toxicity [69]. Understanding this specific relationship could open new avenues in PD treatment, with a focus on preventing TOM40 degradation.

Our research also highlights a significant correlation between α-Syn accumulation and mtDNA damage, suggesting a direct impact on mitochondrial dysfunction. In the current study, we observed notable accumulation of mtDNA damage, particularly in response to the expression of both WT and MTS α-Syn. Furthermore, under WT α-Syn expressing conditions, we identified distinctive mtDNA mutations in hotspots associated with complex I respiratory chain subunits. This process aligns with the observed increase in mtDNA damage and mitochondrial dysfunction in PD-affected human brains [70, 71]. This relationship is critical, as it links the molecular pathology of α-Syn with the functional decline of mitochondria, evidenced by respiratory defects and altered membrane potential. These findings provide a broader perspective on how α-Syn contributes to neuronal dysfunction in PD.

The strong association between mitochondrial accumulation of α-Syn and impaired energy metabolism has been well-established [4]. Our findings suggest that α-Syn primarily disrupts the TOM complex at the outer mitochondrial membrane, likely through aggregation and interference with TOM40’s function, rather than facilitating its own translocation into mitochondria. In line with earlier discoveries, a promising aspect of our study is the observation that moderate TOM40 supplementation can help in the maintenance of proper mitochondrial import and protein turnover, which could partially ameliorate the deficits in mitochondrial bioenergetics induced by α-Syn. This observation opens the prospect of employing TOM40 restoration as a potential therapeutic strategy. However, the translation of this approach into a clinical setting demands a deeper understanding of the long-term effects of TOM40 supplementation and potential challenges in delivery mechanisms. This is particularly crucial given recent studies demonstrating that elevated TOM40 expression leads to caspase-dependent cell death and neurodegeneration in neuronal eye tissue [72].

Apart from TOM40 supplementation, our findings indicate that inhibiting PARP restores TOM40 levels, offering a second, independent avenue to counteract mitochondrial dysfunction and prevent cell death caused by pathogenic α-Syn accumulation. Nevertheless, it is crucial to note that prolonged PARP inhibition may interference with DNA repair pathways, leading to adverse secondary effects such as myeloid leukemia, which could significantly burden the patient’s condition [73]. Considering this, our research, identified two independent and equally effective approaches to prevent α-Syn-induced mitochondrial defects and cell death, suggests the possibility of developing a combinatorial approach. This approach holds the potential to allow for the reduction of doses of both PARPi and TOM40 supplementation to subtoxic, more tolerable levels. The goal is not only to enhance therapeutic efficacy but also to minimize potential side effects associated with higher doses of each treatment. Future studies should focus on exploring this combinatorial therapy in more detail using in vivo PD models.

In summary, our study establishes a direct link between α-Syn accumulation, TOM40 degradation, and mitochondrial dysfunction in Guam PD. These findings not only advance our understanding of Parkinson’s disease pathology, but also open new potential therapeutic strategies. Future research should prioritize in vivo studies, concentrating on investigating the long-term effects of TOM40 supplementation and PARP inhibition as potential therapies. Our findings represent a substantial step forward in understanding the complex interplay of α-Syn pathology, mitochondrial dysfunction, and neuronal damage in PD, setting the foundation for the development of targeted and effective treatments.

## Supplementary information

Supplementary Figures, Tables, and Original Files

## Data Availability

Cell lines and plasmids generated in this research can be obtained from the corresponding author upon completion of a Houston Methodist Materials Transfer Agreement. The datasets generated and analyzed during the current study are available from the corresponding author upon reasonable request. The mitochondrial DNA sequencing data (Fig. 5E) have been submitted to Genome variation map (https://ngdc.cncb.ac.cn/gvm/) and is accessible through GVM accession number GVM000911.

## References

[1] Zambon F, Cherubini M, Fernandes HJR, Lang C, Ryan BJ, Volpato V, et al. Cellular α-synuclein pathology is associated with bioenergetic dysfunction in Parkinson’s iPSC-derived dopamine neurons. Hum Mol Genet. 2019;28:2001–13. 30753527 10.1093/hmg/ddz038 PMC6548224

[2] Devi L, Raghavendran V, Prabhu BM, Avadhani NG, Anandatheerthavarada HK. Mitochondrial import and accumulation of alpha-synuclein impair complex I in human dopaminergic neuronal cultures and Parkinson disease brain. J Biol Chem. 2008;283:9089–9100. 18245082 10.1074/jbc.M710012200 PMC2431021

[3] Subramaniam SR, Vergnes L, Franich NR, Reue K, Chesselet MF. Region specific mitochondrial impairment in mice with widespread overexpression of alpha-synuclein. Neurobiol Dis. 2014;70:204–13. 25016198 10.1016/j.nbd.2014.06.017 PMC4205109

[4] Ganjam GK, Bolte K, Matschke LA, Neitemeier S, Dolga AM, Höllerhage M, et al. Mitochondrial damage by α-synuclein causes cell death in human dopaminergic neurons. Cell Death Dis. 2019;10:865. 31727879 10.1038/s41419-019-2091-2 PMC6856124

[5] Chinta SJ, Mallajosyula JK, Rane A, Andersen JK. Mitochondrial alpha-synuclein accumulation impairs complex I function in dopaminergic neurons and results in increased mitophagy in vivo. Neurosci Lett. 2010;486:235–9. 20887775 10.1016/j.neulet.2010.09.061 PMC2967673

[6] Li N, Ragheb K, Lawler G, Sturgis J, Rajwa B, Melendez JA, et al. Mitochondrial complex I inhibitor rotenone induces apoptosis through enhancing mitochondrial reactive oxygen species production. J Biol Chem. 2003;278:8516–25. 12496265 10.1074/jbc.M210432200

[7] Flones IH, Fernandez-Vizarra E, Lykouri M, Brakedal B, Skeie GO, Miletic H, et al. Neuronal complex I deficiency occurs throughout the Parkinson’s disease brain, but is not associated with neurodegeneration or mitochondrial DNA damage. Acta Neuropathol. 2018;135:409–25. 29270838 10.1007/s00401-017-1794-7

[8] Park JS, Davis RL, Sue CM. Mitochondrial dysfunction in Parkinson’s disease: new mechanistic insights and therapeutic perspectives. Curr Neurol Neurosci Rep. 2018;18:21. 29616350 10.1007/s11910-018-0829-3 PMC5882770

[9] Dolle C, Flones I, Nido GS, Miletic H, Osuagwu N, Kristoffersen S, et al. Defective mitochondrial DNA homeostasis in the substantia nigra in Parkinson disease. Nat Commun. 2016;7:13548. 27874000 10.1038/ncomms13548 PMC5121427

[10] Reeve AK, Krishnan KJ, Elson JL, Morris CM, Bender A, Lightowlers RN, et al. Nature of mitochondrial DNA deletions in substantia nigra Neurons. Am J Hum Genet. 2008;82:228–35. 18179904 10.1016/j.ajhg.2007.09.018 PMC2253975

[11] Vasquez V, Mitra J, Hegde PM, Pandey A, Sengupta S, Mitra S, et al. Chromatin-bound oxidized alpha-synuclein causes strand breaks in neuronal genomes in in vitro models of Parkinson’s disease. J Alzheimers Dis. 2017;60:S133–s150. 28731447 10.3233/JAD-170342 PMC6172953

[12] Milanese C, Cerri S, Ulusoy A, Gornati SV, Plat A, Gabriels S, et al. Activation of the DNA damage response in vivo in synucleinopathy models of Parkinson’s disease. Cell Death Dis. 2018;9:818. 30050065 10.1038/s41419-018-0848-7 PMC6062587

[13] Shiota T, Imai K, Qiu J, Hewitt VL, Tan K, Shen HH, et al. Molecular architecture of the active mitochondrial protein gate. Science. 2015;349:1544–8. 26404837 10.1126/science.aac6428

[14] Needs HI, Protasoni M, Henley JM, Prudent J, Collinson I, Pereira GC. Interplay between mitochondrial protein import and respiratory complexes assembly in neuronal health and degeneration. Life. 2021;11:432. 34064758 10.3390/life11050432 PMC8151517

[15] Busch JD, Fielden LF, Pfanner N, Wiedemann N. Mitochondrial protein transport: Versatility of translocases and mechanisms. Mol Cell. 2023;83:890–910. 36931257 10.1016/j.molcel.2023.02.020

[16] Rath S, Sharma R, Gupta R, Ast T, Chan C, Durham TJ, et al. MitoCarta3.0: an updated mitochondrial proteome now with sub-organelle localization and pathway annotations. Nucleic Acids Res. 2021;49:D1541–d1547. 33174596 10.1093/nar/gkaa1011 PMC7778944

[17] Wang W, Chen X, Zhang L, Yi J, Ma Q, Yin J, et al. Atomic structure of human TOM core complex. Cell Discov. 2020;6:67. 33083003 10.1038/s41421-020-00198-2 PMC7522991

[18] Pfanner N, Warscheid B, Wiedemann N. Mitochondrial proteins: from biogenesis to functional networks. Nat Rev Mol Cell Biol. 2019;20:267–84. 30626975 10.1038/s41580-018-0092-0 PMC6684368

[19] Baker KP, Schaniel A, Vestweber D, Schatz G. A yeast mitochondrial outer membrane protein essential for protein import and cell viability. Nature. 1990;348:605–9. 2250717 10.1038/348605a0

[20] Milenkovic D, Kozjak V, Wiedemann N, Lohaus C, Meyer HE, Guiard B, et al. Sam35 of the mitochondrial protein sorting and assembly machinery is a peripheral outer membrane protein essential for cell viability. J Biol Chem. 2004;279:22781–5. 15067005 10.1074/jbc.C400120200

[21] Gornicka A, Bragoszewski P, Chroscicki P, Wenz LS, Schulz C, Rehling P, et al. A discrete pathway for the transfer of intermembrane space proteins across the outer membrane of mitochondria. Mol Biol Cell. 2014;25:3999–4009. 25318675 10.1091/mbc.E14-06-1155 PMC4263444

[22] Honea RA, Hunt S, Lepping RJ, Vidoni ED, Morris JK, Watts A, et al. Alzheimer’s disease cortical morphological phenotypes are associated with TOMM40'523-APOE haplotypes. Neurobiol Aging. 2023;132:131–44. 37804609 10.1016/j.neurobiolaging.2023.09.001 PMC10763175

[23] Kulminski AM, Philipp I, Shu L, Culminskaya I. Definitive roles of TOMM40-APOE-APOC1 variants in the Alzheimer’s risk. Neurobiol Aging. 2022;110:122–31. 34625307 10.1016/j.neurobiolaging.2021.09.009 PMC8758518

[24] Sabbagh MN, Pope E, Cordes L, Shi J, DeCourt B. Therapeutic considerations for APOE and TOMM40 in Alzheimers disease: a tribute to Allen Roses MD. Expert Opin Investig Drugs. 2021;30:39–44. 33455481 10.1080/13543784.2021.1849138 PMC9262379

[25] Chen S, Sarasua SM, Davis NJ, DeLuca JM, Boccuto L, Thielke SM, et al. TOMM40 genetic variants associated with healthy aging and longevity: a systematic review. BMC Geriatr. 2022;22:667. 35964003 10.1186/s12877-022-03337-4 PMC9375314

[26] Bakeberg MC, Hoes ME, Gorecki AM, Theunissen F, Pfaff AL, Kenna JE, et al. The TOMM40 ‘523’ polymorphism in disease risk and age of symptom onset in two independent cohorts of Parkinson’s disease. Sci Rep. 2021;11:6363. 33737565 10.1038/s41598-021-85510-0 PMC7973542

[27] Bender A, Desplats P, Spencer B, Rockenstein E, Adame A, Elstner M, et al. TOM40 mediates mitochondrial dysfunction induced by alpha-synuclein accumulation in Parkinson’s disease. PLoS ONE. 2013;8:e62277. 23626796 10.1371/journal.pone.0062277 PMC3633917

[28] Di Maio R, Barrett PJ, Hoffman EK, Barrett CW, Zharikov A, Borah A, et al. alpha-Synuclein binds to TOM20 and inhibits mitochondrial protein import in Parkinson’s disease. Sci Transl Med. 2016;8:342ra378. 10.1126/scitranslmed.aaf3634 PMC5016095 27280685

[29] Koo JH, Cho JY, Lee UB. Treadmill exercise alleviates motor deficits and improves mitochondrial import machinery in an MPTP-induced mouse model of Parkinson’s disease. Exp Gerontol. 2017;89:20–29. 28062370 10.1016/j.exger.2017.01.001

[30] Mekhaeil M, Dev KK, Conroy MJ. Existing evidence for the repurposing of PARP-1 inhibitors in rare demyelinating diseases. Cancers. 2022;14:687. 10.3390/cancers14030687 PMC8833351 35158955

[31] Kam TI, Mao X, Park H, Chou SC, Karuppagounder SS, Umanah GE, et al. Poly(ADP-ribose) drives pathologic alpha-synuclein neurodegeneration in Parkinson’s disease. Science. 2018;362:eaat8407. 10.1126/science.aat8407 PMC6431793 30385548

[32] Kang M, Park S, Park SH, Lee HG, Park JH. A double-edged sword: the two faces of PARylation. Int J Mol Sci. 2022;23:9826. 10.3390/ijms23179826 PMC9456079 36077221

[33] Mao K, Chen J, Yu H, Li H, Ren Y, Wu X, et al. Poly (ADP-ribose) polymerase 1 inhibition prevents neurodegeneration and promotes α-synuclein degradation via transcription factor EB-dependent autophagy in mutant α-synucleinA53T model of Parkinson’s disease. Aging Cell. 2020;19:e13163. 32475059 10.1111/acel.13163 PMC7294777

[34] Plato CC, Galasko D, Garruto RM, Plato M, Gamst A, Craig UK, et al. ALS and PDC of Guam: forty-year follow-up. Neurology. 2002;58:765–73. 11889241 10.1212/wnl.58.5.765

[35] Mitra J, Guerrero EN, Hegde PM, Liachko NF, Wang H, Vasquez V, et al. Motor neuron disease-associated loss of nuclear TDP-43 is linked to DNA double-strand break repair defects. Proc Natl Acad Sci USA. 2019;116:4696–705. 30770445 10.1073/pnas.1818415116 PMC6410842

[36] Rhinn H, Qiang L, Yamashita T, Rhee D, Zolin A, Vanti W, et al. Alternative alpha-synuclein transcript usage as a convergent mechanism in Parkinson’s disease pathology. Nat Commun. 2012;3:1084. 23011138 10.1038/ncomms2032 PMC3660047

[37] Dettmer U, Newman AJ, Luth ES, Bartels T, Selkoe D. In Vivo cross-linking reveals principally oligomeric forms of α-synuclein and β-synuclein in neurons and non-neural cells. J Biol Chem. 2013;288:6371–85. 23319586 10.1074/jbc.M112.403311 PMC3585072

[38] Dimauro I, Pearson T, Caporossi D, Jackson MJ. A simple protocol for the subcellular fractionation of skeletal muscle cells and tissue. BMC Res Notes. 2012;5:513. 22994964 10.1186/1756-0500-5-513 PMC3508861

[39] Kodavati M, Wang H, Guo W, Mitra J, Hegde PM, Provasek V, et al. FUS unveiled in mitochondrial DNA repair and targeted ligase-1 expression rescues repair-defects in FUS-linked motor neuron disease. Nat Commun. 2024;15:2156. 38461154 10.1038/s41467-024-45978-6 PMC10925063

[40] Schmittgen TD, Livak KJ. Analyzing real-time PCR data by the comparative C(T) method. Nat Protoc. 2008;3:1101–8. 18546601 10.1038/nprot.2008.73

[41] Soubannier V, McLelland GL, Zunino R, Braschi E, Rippstein P, Fon EA, et al. A vesicular transport pathway shuttles cargo from mitochondria to lysosomes. Curr Biol. 2012;22:135–41. 22226745 10.1016/j.cub.2011.11.057

[42] Sugiura A, McLelland GL, Fon EA, McBride HM. A new pathway for mitochondrial quality control: mitochondrial-derived vesicles. EMBO J. 2014;33:2142–56. 25107473 10.15252/embj.201488104 PMC4282503

[43] Gu X, Ma Y, Liu Y, Wan Q. Measurement of mitochondrial respiration in adherent cells by Seahorse XF96 Cell Mito Stress Test. STAR Protoc. 2021;2:100245. 33458707 10.1016/j.xpro.2020.100245 PMC7797920

[44] Dasgupta S, Koch R, Westra WH, Califano JA, Ha PK, Sidransky D, et al. Mitochondrial DNA mutation in normal margins and tumors of recurrent head and neck squamous cell carcinoma patients. Cancer Prev Res. 2010;3:1205–11. 10.1158/1940-6207.CAPR-10-0018 PMC3040952 20660573

[45] Das BC, Dasgupta S, Ray SK. Potential therapeutic roles of retinoids for prevention of neuroinflammation and neurodegeneration in Alzheimer’s disease. Neural Regen Res. 2019;14:1880–92. 31290437 10.4103/1673-5374.259604 PMC6676868

[46] Cole NB, DiEuliis D, Leo P, Mitchell DC, Nussbaum RL. Mitochondrial translocation of α-synuclein is promoted by intracellular acidification. Exp Cell Res. 2008;314:2076–89. 18440504 10.1016/j.yexcr.2008.03.012 PMC2483835

[47] Koss DJ, Erskine D, Porter A, Palmoski P, Menon H, Todd OGJ, et al. Nuclear alpha-synuclein is present in the human brain and is modified in dementia with Lewy bodies. Acta Neuropathol Commun. 2022;10:98. 35794636 10.1186/s40478-022-01403-x PMC9258129

[48] Lee HJ, Choi C, Lee SJ. Membrane-bound alpha-synuclein has a high aggregation propensity and the ability to seed the aggregation of the cytosolic form. J Biol Chem. 2002;277:671–8. 11679584 10.1074/jbc.M107045200

[49] Mishra A, Krishnamurthy S. Rebamipide mitigates impairments in mitochondrial function and bioenergetics with α-synuclein pathology in 6-OHDA-induced Hemiparkinson’s Model in rats. Neurotox Res. 2019;35:542–62. 30610666 10.1007/s12640-018-9983-2

[50] Flierl A, Oliveira LM, Falomir-Lockhart LJ, Mak SK, Hesley J, Soldner F, et al. Higher vulnerability and stress sensitivity of neuronal precursor cells carrying an alpha-synuclein gene triplication. PLoS ONE. 2014;9:e112413. 25390032 10.1371/journal.pone.0112413 PMC4229205

[51] Byers B, Cord B, Nguyen HN, Schüle B, Fenno L, Lee PC, et al. SNCA triplication Parkinson’s patient’s iPSC-derived DA neurons accumulate α-synuclein and are susceptible to oxidative stress. PLoS ONE. 2011;6:e26159. 22110584 10.1371/journal.pone.0026159 PMC3217921

[52] Zhang Y, Sanner MF. AutoDock CrankPep: combining folding and docking to predict protein-peptide complexes. Bioinformatics. 2019;35:5121–7. 31161213 10.1093/bioinformatics/btz459 PMC6954657

[53] Vasquez V, Mitra J, Perry G, Rao KS, Hegde ML. An inducible alpha-synuclein expressing neuronal cell line model for Parkinson’s disease1. J Alzheimers Dis. 2018;66:453–60. 30320583 10.3233/JAD-180610 PMC6221916

[54] Emin D, Zhang YP, Lobanova E, Miller A, Li X, Xia Z, et al. Small soluble α-synuclein aggregates are the toxic species in Parkinson’s disease. Nat Commun. 2022;13:5512. 36127374 10.1038/s41467-022-33252-6 PMC9489799

[55] Song J, Herrmann JM, Becker T. Quality control of the mitochondrial proteome. Nat Rev Mol Cell Biol. 2021;22:54–70. 33093673 10.1038/s41580-020-00300-2

[56] Vazquez-Calvo C, Suhm T, Büttner S, Ott M. The basic machineries for mitochondrial protein quality control. Mitochondrion. 2020;50:121–31. 31669238 10.1016/j.mito.2019.10.003

[57] Lei Z, Cao G, Wei G. A30P mutant α-synuclein impairs autophagic flux by inactivating JNK signaling to enhance ZKSCAN3 activity in midbrain dopaminergic neurons. Cell Death Dis. 2019;10:133. 30755581 10.1038/s41419-019-1364-0 PMC6372582

[58] Youn J, Lee S-B, Lee HS, Yang HO, Park J, Kim JS, et al. Cerebrospinal fluid levels of autophagy-related proteins represent potentially novel biomarkers of early-stage Parkinson’s disease. Sci Rep. 2018;8:16866. 30442917 10.1038/s41598-018-35376-6 PMC6237988

[59] Towers CG, Wodetzki DK, Thorburn J, Smith KR, Caino MC, Thorburn A. Mitochondrial-derived vesicles compensate for loss of LC3-mediated mitophagy. Dev Cell. 2021;56:2029–2042.e2025. 34171288 10.1016/j.devcel.2021.06.003 PMC8319140

[60] Liang W, Sagar S, Ravindran R, Najor RH, Quiles JM, Chi L, et al. Mitochondria are secreted in extracellular vesicles when lysosomal function is impaired. Nat Commun. 2023;14:5031. 37596294 10.1038/s41467-023-40680-5 PMC10439183

[61] Chabot P, Hsia TC, Ryu JS, Gorbunova V, Belda-Iniesta C, Ball D, et al. Veliparib in combination with whole-brain radiation therapy for patients with brain metastases from non-small cell lung cancer: results of a randomized, global, placebo-controlled study. J Neurooncol. 2017;131:105–15. 27655223 10.1007/s11060-016-2275-x PMC5258788

[62] Blandini F, Armentero MT, Martignoni E. The 6-hydroxydopamine model: news from the past. Parkinsonism Relat Disord. 2008;14:S124–129. 18595767 10.1016/j.parkreldis.2008.04.015

[63] Wang X, Becker K, Levine N, Zhang M, Lieberman AP, Moore DJ, et al. Pathogenic alpha-synuclein aggregates preferentially bind to mitochondria and affect cellular respiration. Acta Neuropathol Commun. 2019;7:41. 30871620 10.1186/s40478-019-0696-4 PMC6419482

[64] Liao PC, Wolken DMA, Serrano E, Srivastava P, Pon LA. Mitochondria-associated degradation pathway (MAD) function beyond the outer membrane. Cell Rep. 2020;32:107902. 32668258 10.1016/j.celrep.2020.107902 PMC7391283

[65] Lavie J, De Belvalet H, Sonon S, Ion AM, Dumon E, Melser S, et al. Ubiquitin-dependent degradation of mitochondrial proteins regulates energy metabolism. Cell Rep. 2018;23:2852–63. 29874573 10.1016/j.celrep.2018.05.013

[66] Wang S, Long H, Hou L, Feng B, Ma Z, Wu Y, et al. The mitophagy pathway and its implications in human diseases. Signal Transduct Target Ther. 2023;8:304. 37582956 10.1038/s41392-023-01503-7 PMC10427715

[67] Okatsu K, Kimura M, Oka T, Tanaka K, Matsuda N. Unconventional PINK1 localization to the outer membrane of depolarized mitochondria drives Parkin recruitment. J Cell Sci. 2015;128:964–78. 25609704 10.1242/jcs.161000 PMC4342580

[68] Kinnart I, Manders L, Heyninck T, Imberechts D, Praschberger R, Schoovaerts N, et al. Elevated α-synuclein levels inhibit mitophagic flux. npj Parkinson’s Dis. 2024;10:80. 38594264 10.1038/s41531-024-00696-0 PMC11004019

[69] De Miranda BR, Rocha EM, Castro SL, Greenamyre JT. Protection from α-Synuclein induced dopaminergic neurodegeneration by overexpression of the mitochondrial import receptor TOM20. npj Parkinson’s Dis. 2020;6:38. 33293540 10.1038/s41531-020-00139-6 PMC7722884

[70] Nido GS, Dolle C, Flones I, Tuppen HA, Alves G, Tysnes OB, et al. Ultradeep mapping of neuronal mitochondrial deletions in Parkinson’s disease. Neurobiol Aging. 2018;63:120–7. 29257976 10.1016/j.neurobiolaging.2017.10.024

[71] Reeve AK, Grady JP, Cosgrave EM, Bennison E, Chen C, Hepplewhite PD, et al. Mitochondrial dysfunction within the synapses of substantia nigra neurons in Parkinson’s disease. npj Parkinsons Dis. 2018;4:9. 29872690 10.1038/s41531-018-0044-6 PMC5979968

[72] Periasamy A, Mitchell N, Zaytseva O, Chahal AS, Zhao J, Colman PM, et al. An increase in mitochondrial TOM activates apoptosis to drive retinal neurodegeneration. Sci Rep. 2022;12:21634. 36517509 10.1038/s41598-022-23280-z PMC9750964

[73] LaFargue CJ, Dal Molin GZ, Sood AK, Coleman RL. Exploring and comparing adverse events between PARP inhibitors. Lancet Oncol. 2019;20:e15–e28. 30614472 10.1016/S1470-2045(18)30786-1 PMC7292736

