# Supplementary material for: Mitochondria-targeted oligomeric α-synuclein induces TOM40 degradation and mitochondrial dysfunction in Parkinson’s disease and parkinsonism-dementia of Guam
Source: Cell Death Dis. 2024 Dec 18;15(12):914. doi: 10.1038/s41419-024-07258-5 (PMC11655978; doi:10.1038/s41419-024-07258-5)
Supplement: Supplementary file 1 — Supplementary Figures, Tables, and Original Files [file 41419_2024_7258_MOESM1_ESM.pdf]

Supplementary Information

Mitochondria-Targeted Oligomeric α-Synuclein Induces TOM40 Degradation and Mitochondrial Dysfunction in Parkinson's Disease and Parkinsonism-Dementia of Guam

Velmarini Vasquez [a,b#], Manohar Kodavati [a#], Joy Mitra [a], Indira Vedula [c], Dale J. Hamilton [c, d], Ralph M. Garruto [e], K.S. Rao [f], and Muralidhar L. Hegde [a, g*]

[a] Division of DNA Repair Research, Center for Neuroregeneration, Department of Neurosurgery, Houston Methodist Research Institute, Houston, TX, USA.

[b] Neuroscience Center, Instituto de Investigaciones Científicas y Servicios de Alta Tecnología, (INDICASAT AIP), Panama City, Panama.

[c] Center for Bioenergetics, Houston Methodist Research Institute, Houston, TX, USA.

[d] Department of Medicine, Houston Methodist, Weill Cornell Medicine affiliate, Houston, TX, USA.

[e] Departments of Anthropology and Biological Sciences, Binghamton University, State University of New York, Binghamton, NY, USA.

[f] Department of Biotechnology, KLEF Deemed to be University, Vaddeswaram, India.

[g] Department of Neuroscience, Weill Cornell Medical College, NY, USA.

[#] These authors contributed equally

**Corresponding authors:* Muralidhar L. Hegde, PhD, Email: mlhegde@houstonmethodist.org;

Communicating Autor Address:
MURALIDHAR L. HEGDE, *Ph.D.*
Professor, Department of Neurosurgery
Everett E. and Randee K. Bernal Centennial Endowed Chair of Neurological Institute
Director, Division of DNA Repair Research
Center for Neuroregeneration
Houston Methodist Research Institute
6670 Bertner Ave, RI-11-112
Houston, Texas 77030, USA
713-441-7456 (O) I 409-457-7079 (C)
Email: mlhegde@houstonmethodist.org

Supplementary information accompanying this manuscript contains four figures and six tables, as well as supplementary files containing original Western Blot images and raw qPCR data.

Supplementary Figures

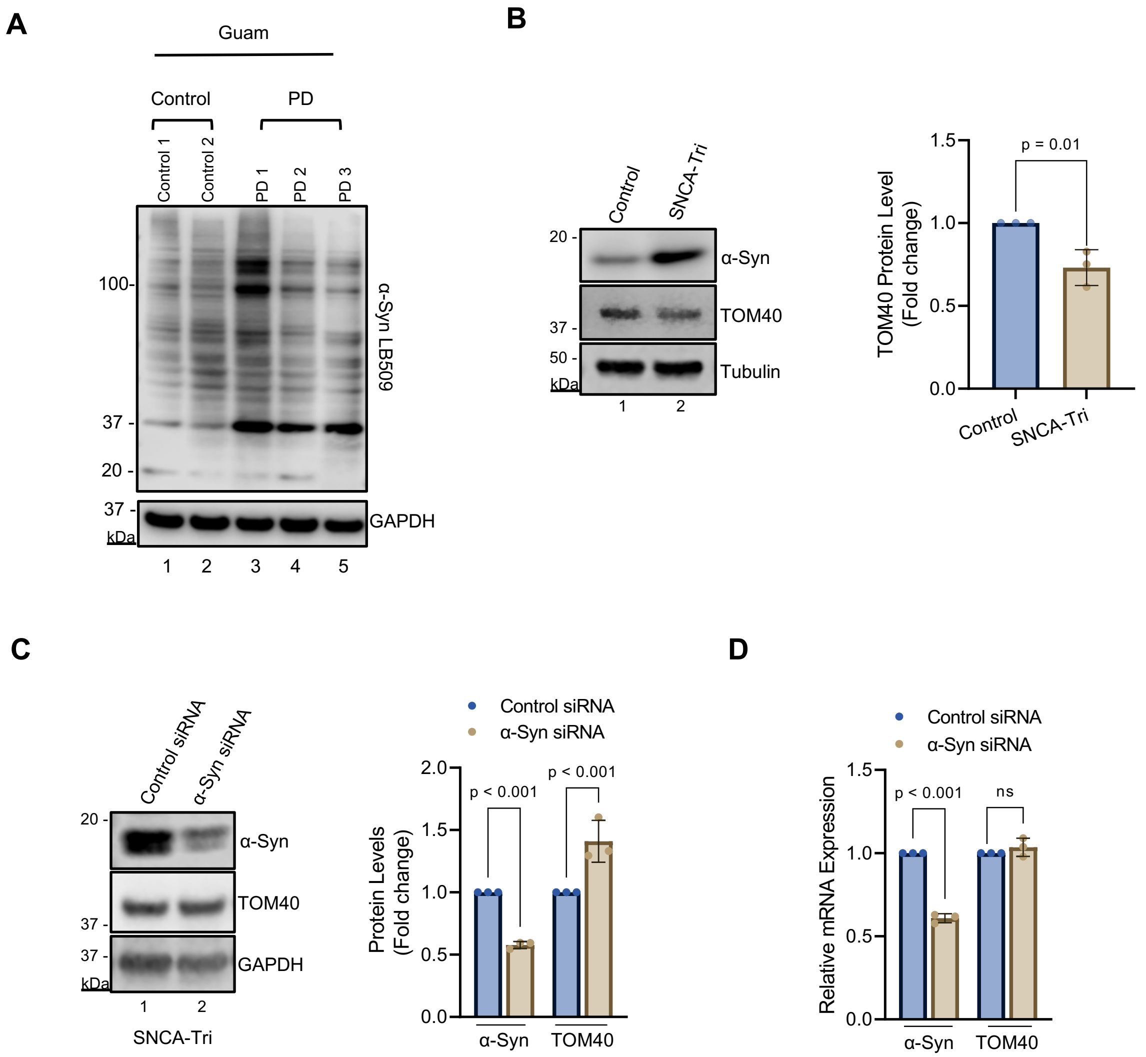

Supplementary Fig. 1. A (Related to Fig. 1A). α-Syn protein expression in Guam PD patient brain tissue**.** Representative immunoblotting of Guam PD and Guam non-neurological controls brain tissue extracts demonstrates increased α-Syn protein levels (Lns 3-5). **B (Related to Fig. 1D).** Representative immunoblot of whole cell extracts and densitometry analysis revealing reduced TOM40 levels (Ln 2) in SNCA-Tri line. **C (Related to Fig. 1E).** Representative immunoblotting of whole-cell extracts from SNCA-Tri cells following siRNA mediated α-Syn knockdown, confirming reduced α-Syn protein expression while TOM40 protein levels remain stable (Ln 2). **D (Related to Fig. 1E).** RT-qPCR revealing α-Syn knockdown does not affect TOM40 mRNA expression in the SNCA-Tri line. Data are presented as mean ± s.e.m. from three independent experiments. Statistical analysis was performed using student's t-test (**B**) and two-way ANOVA (**C, D**). ns = non-significant ($p > 0.05$).

Supplementary Figures

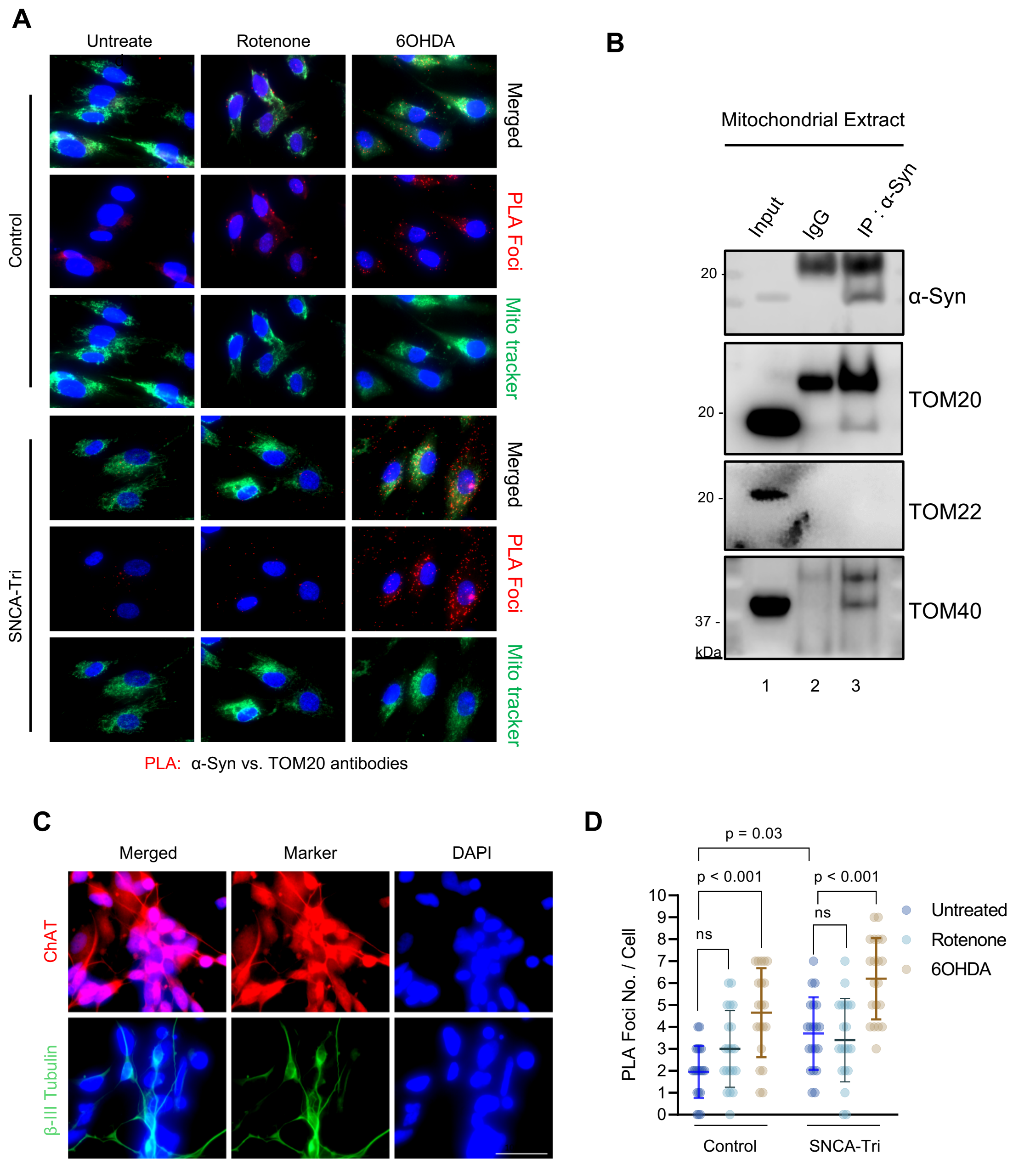

Supplementary Fig. 2 (Related to Fig. 2E-F). A. Individual channels for PLA images highlighting the interaction between α-Syn and TOM20 (red foci), co-stained with MitoTracker (green). Scale bar = 10 µm. **B.** Immunoblot of *in vitro* recombinant α-Syn interaction with mitochondrial outer membrane proteins in isolated mitochondria from NPSCs. **C.** Differentiated NPSCs utilized for PLA analysis characterized by a high number of choline acetyltransferase (ChAT) and beta tubulin III (β-III Tubulin) positive cells, definitive markers for cholinergic, and neuronal differentiation, respectively. Scale bar = 10 µm. **D.** PLA analysis indicating a significant difference in PLA foci counts between Control and SNCA-Tri NPSCs following 6OHDA and Rotenone exposure in differentiated cortical neurons. Quantification of PLA foci was derived from 50 cells and statistical analysis was performed using one-way ANOVA. ns = non-significant ($p > 0.05$).

Supplementary Figures

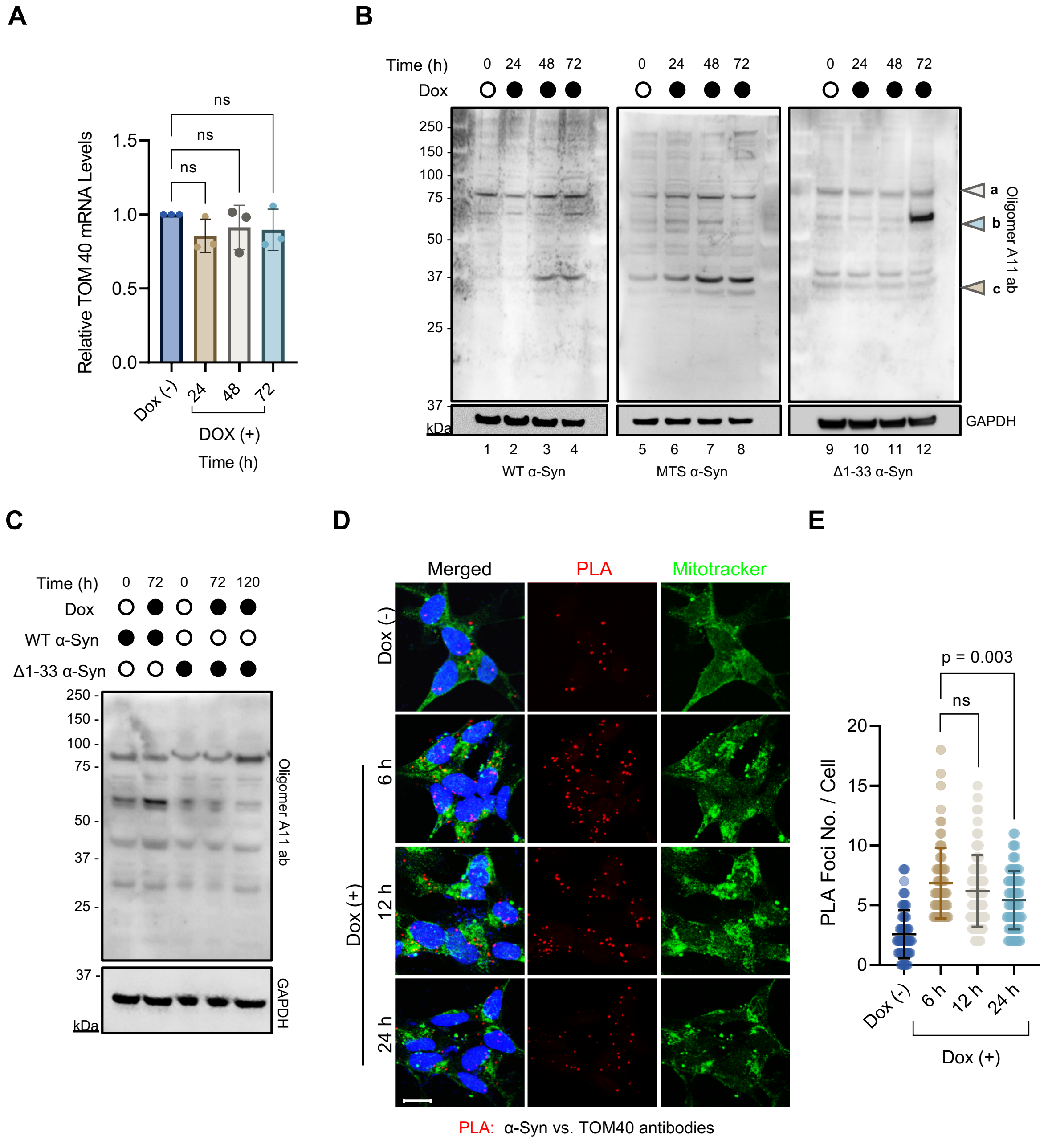

Supplementary Fig. 3. A (Related to Fig. 3C). RT-qPCR revealed that WT α-Syn overexpression does not impact TOM40 mRNA levels. Data are presented as mean ± s.e.m. from three independent experiments. Statistical analysis was performed using one-way ANOVA. **B (Related to Fig. 3C).** Representative immunoblot of whole cell lysate extracts: Abundance of α-Syn oligomer conformations (Lns 2-4, 6-8, 12) indicated by (a) 100-75 kDa, (b) 50-75 kDa, and (c) 37 kDa. **C (Related to Fig. 3C).** Representative immunoblot of extended Dox induced Δ1-33 α-Syn expression time showed a comparable 100-75 kDa band to the 72 hours WT α-Syn expression, only after 120 hours Δ1-33 α-Syn expression. **D-E (Related to Fig. 3G).** PLA demonstrates that α-Syn interacts with TOM40 at early Dox induced α-Syn timepoints. Quantification of PLA foci was derived from 75 cells and statistical analysis was performed using one-way ANOVA. ns = non-significant ($p > 0.05$).

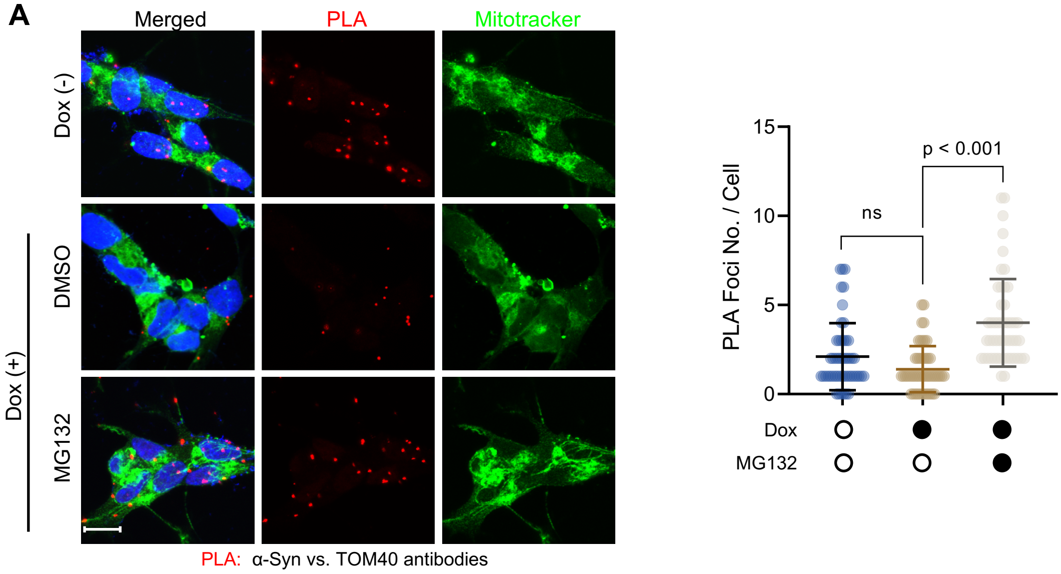

Supplementary Fig. 4 (Related to Fig. 4B). A. Inhibiting TOM40 degradation increases α-Syn interaction with TOM40 after 48 hours of α-Syn Dox induced expression. Statistical analysis was performed using one-way ANOVA. ns = non-significant ($p > 0.05$).

Supplementary Table 1. Demographic details of Guamanian PD, ALS, and non-neurological control brain tissues from the Binghamton Biospecimen Archive.

Sample ID	Sex	Age (Years)	Neural Fibrillary Tangle Status
**Control 1**	M	49	Moderate
**Control 2**	F	57	Absent
**Control 3**	F	43	Absent
**PD 1**	M	62	Severe
**PD 2**	M	69	Severe
**PD 3**	M	72	Moderate
**PD 4**	M	66	Moderate
**ALS 1**	F	54	Moderate
**ALS 2**	F	47	Severe
**ALS 3**	M	45	Severe

Supplementary Table 2. Antibodies used for immunoblotting (IB), immunofluorescence (IF), and proximity ligation assay (PLA).

Antibody (Ab) Name	Manufacturer (Reference Number)	Species	Technique (Dilution)
TOM40	Proteintech (18409-1 AP)	Rabbit	IB (1:1000), IF & PLA (1:100)
TOM20	Santa Cruz (FL-145)	Rabbit	IB (1:1000), IF & PLA (1:100)
TOM22	Santa Cruz (SC-58308)	Mouse	IB (1:1000)
α-Syn AP	Proteintech (10842-1-AP)	Rabbit	IB (1:1000)
α-Syn 3H2897	Santa Cruz (SC-69977)	Mouse	IF & PLA (1:25)
α-Syn LB509	Santa Cruz (SC-58480)	Mouse	IB (1:500)
α-Syn 204	Biolegend (838201)	Mouse	IB (1:1000)
α-Syn EP1646	Millipore (04-1053)	Rabbit	IB (1:1000)
Oligomer A11	Thermo Fisher (AHB0052)	Rabbit	IB (1:1000)
Monoclonal ANTI-FLAG M2-Peroxidase	Sigma (A8592)	Mouse	IB (1:1000)
Anti-DDDDK tag	Abcam (ab1162)	Rabbit	PLA (1:300)
Anti LC3B	Abcam (ab51520)	Rabbit	IB (1:2000)
Anti-Ubiquitin	Sigma Aldrich ST1200	Mouse	IB (1:1000)
Poly / Mono ADP Ribose E6F6A	CellSignaling (837325)	Rabbit	IB (1:1000)
GAPDH	Novusbio (NB615)	Mouse	IB (1:3000)
β-Actin [GT5512]	GeneTex (GTX629630)	Mouse	IB (1:3000)
Tubulin [YL1/2]	Abcam (ab6160)	Rat	IB (1:3000)
Nestin	Genetex (GTX30671)	Mouse	IF (1:100)

Supplementary Table 3. Real-time PCR primer sequences used in Fig.1 and Supplementary. Fig. 3.

Name	Primer Sequence	Amplicon Size (bp)
TOM40	**Fwd:** AGGAATCCTCGTAGCCCACT<br>**Rev:** CTGGTGTCCTGCATCCTTGT	252
α-SynT	**Fwd:** AGGGTGTTCTCTATGTAGG<br>**Rev:** ACTGTCTTCTGGGCTACTGC	215
TOM20	**Fwd:** AGAGCTGGGCTTTCCAAGTTAC<br>**Rev:** GGTGGTGGAAGAGTTTGCTGT	200
HPRT	**Fwd:** TGACCTTGATTTATTTTGCATACC<br>**Rev:** CGAGCAAGACGTTCAGTCCT	198

Supplementary Table 4. Primers for generating inducible Flag α-Syn plasmid constructs in Fig. 3C.

Name	Primer Sequence	Amplicon Size (bp)
WT-α-Syn	**Fwd:** GCGACCATGGATGGATGTATTCATGAAAGGACT<br>**Rev:** TCGTGTCGACTTAGGCTTCAGGTTCGTAGTCTTGATACC	420
pCW WT α-Syn-Flag	**Fwd:** CTCTGCTAGCATGGATGTATTCATGAAAGGACT<br>**Rev:** CACTGTCGACTTACTTATCGTCATCGTCTTTG TAATCGGCTTCAGGTTCGTAGTCTTGATACC	428
pCW ∆1-33 α-Syn-Flag	**Fwd:** CTCTGCTAGCATGAAAGAGGGTGTTCTCTATGTAGGC<br>**Rev:** CACTGTCGACTTACTTATCGTCATCGTCTTTGTAATCGGCT TCAGGTTCGTAGTCTTGATACC	326
pCW MTS α-Syn-Flag	**Fwd:** TAAGGCTAGCATGTCCGTCCTGACGCCGCTGCT<br>**Rev:** CACTGTCGACTTACTTATCGTCATCGTCTTTGTAATCGGCTT CAGGTTCGTAGTCTTGATACC	528
COX2 MTS	**Sense:**<br>CTAGAATGTCCGTCCTGACGCCGCTGCTGGGCTTGACAGGCTCG GCCCGGCGGCTCCCAGTGCCGCGCGCCAAGATCCATTCGTTGC<br>**Antisense:**<br>CATGGCAACGAATGGATCTTGGCGCGCGGCACTGGGAGCCGCC GGGCCGAGCCTGTCAAGCCCCGCAGCAGCAGCGGCGTCAGGAC GGACATT	93

Supplementary Table 5. Mitochondrial LA-PCR primer sequences used in Fig. 5.

Name	Primer Sequence	Amplicon Size
mtLA 5999-1481	**Fwd:** TGGGATTACACGTGTGAACCAACC<br>**Rev:** GCTCTACCCTCCCTCTACCGTCC	10.4 Kbp
mtLA 7601-16401	**Fwd:** CAAGTAGGTCTACAAGACG<br>**Rev:** GGATATTGATTTCACGGAGG	10 Kbp
mtLA 179-9231	**Fwd:** TTACAGGCGAACATACTTAC<br>**Rev:** GATAGGCATGTGATTGGTG	10 Kbp
mtSA ND1	**Fwd:** AAGAACACCTCTGATTACTCCTGCC<br>**Rev:** GTTGTGTAGAGTTCAGGGGAGAGTGCG	250 bp

Supplementary Table 6. Nature of mitochondrial DNA mutations in α-Syn expressing cells. Related to Fig. 5C.

Mutation type	WT α-Syn Dox (-)	WT α-Syn Dox (+)
A/AC	31	32
A/AT	5	6
A/C	0	2
A/G	9	9
A/T	1	1
C/A	2	1
C/CA	16	16
C/CG	1	1
C/CT	4	5
C/T	6	7
CN/C	1	1
CT/C	0	1
G/A	12	17
G/GA	12	12
G/GC	14	14
G/GT	1	1
G/T	0	1
T/A	0	2
T/C	5	5
T/G	1	2
T/TA	11	12
T/TC	14	16
T/TG	2	3

Supplementary File 1

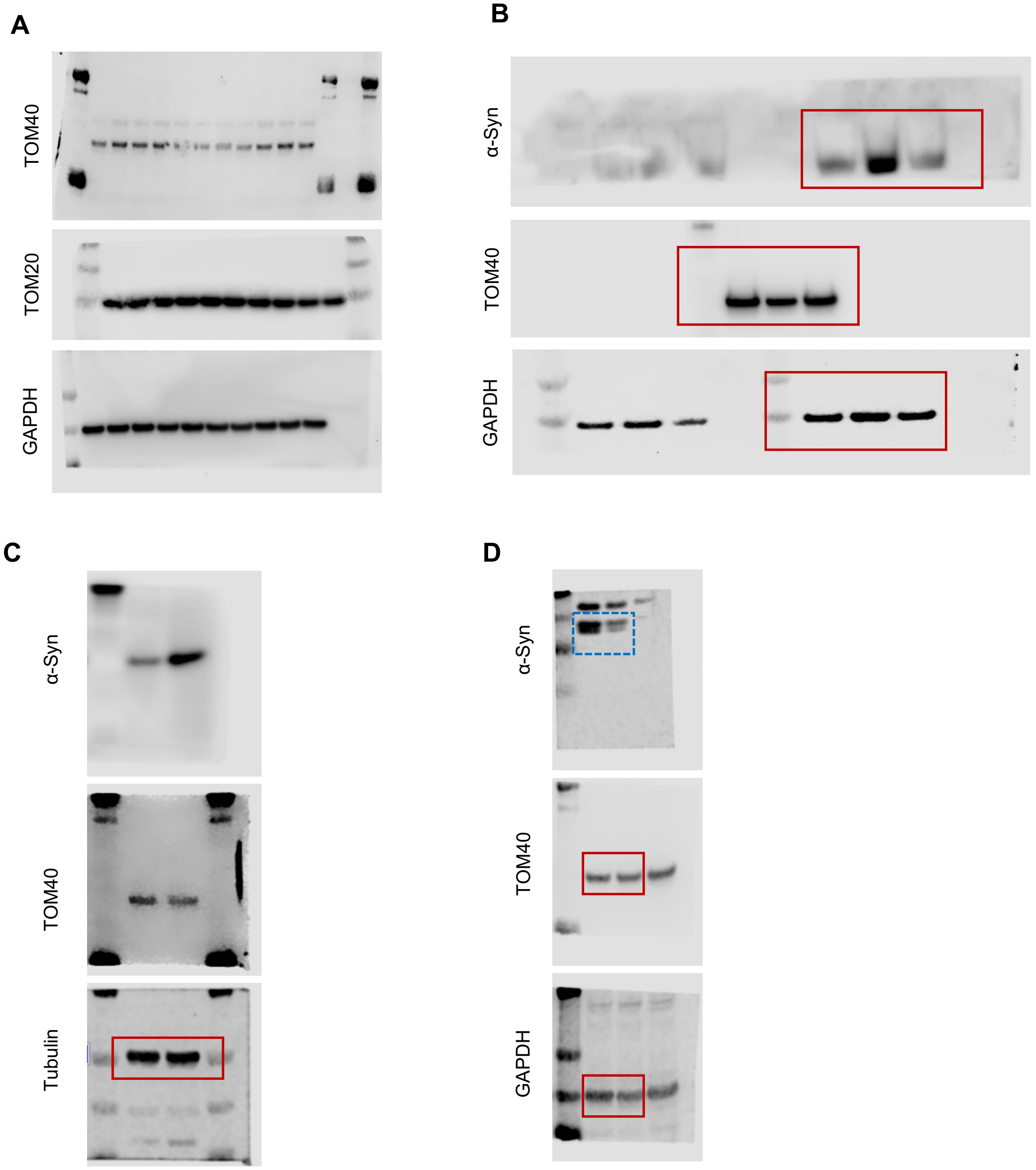

Supplementary File 1. A. Original Western Blot images for Fig. 1A Fig**. B.** Original Western Blot images for Fig. 1F. **C.** Original Western Blot images for Fig.Supplementary Fig 1B. **D.** Original Western Blot images for Fig.Supplementary 1C. The red box delineates the lanes selected for inclusion in the final representative image. The dashed blue box delineates the protein band of interest.

Supplementary File 2

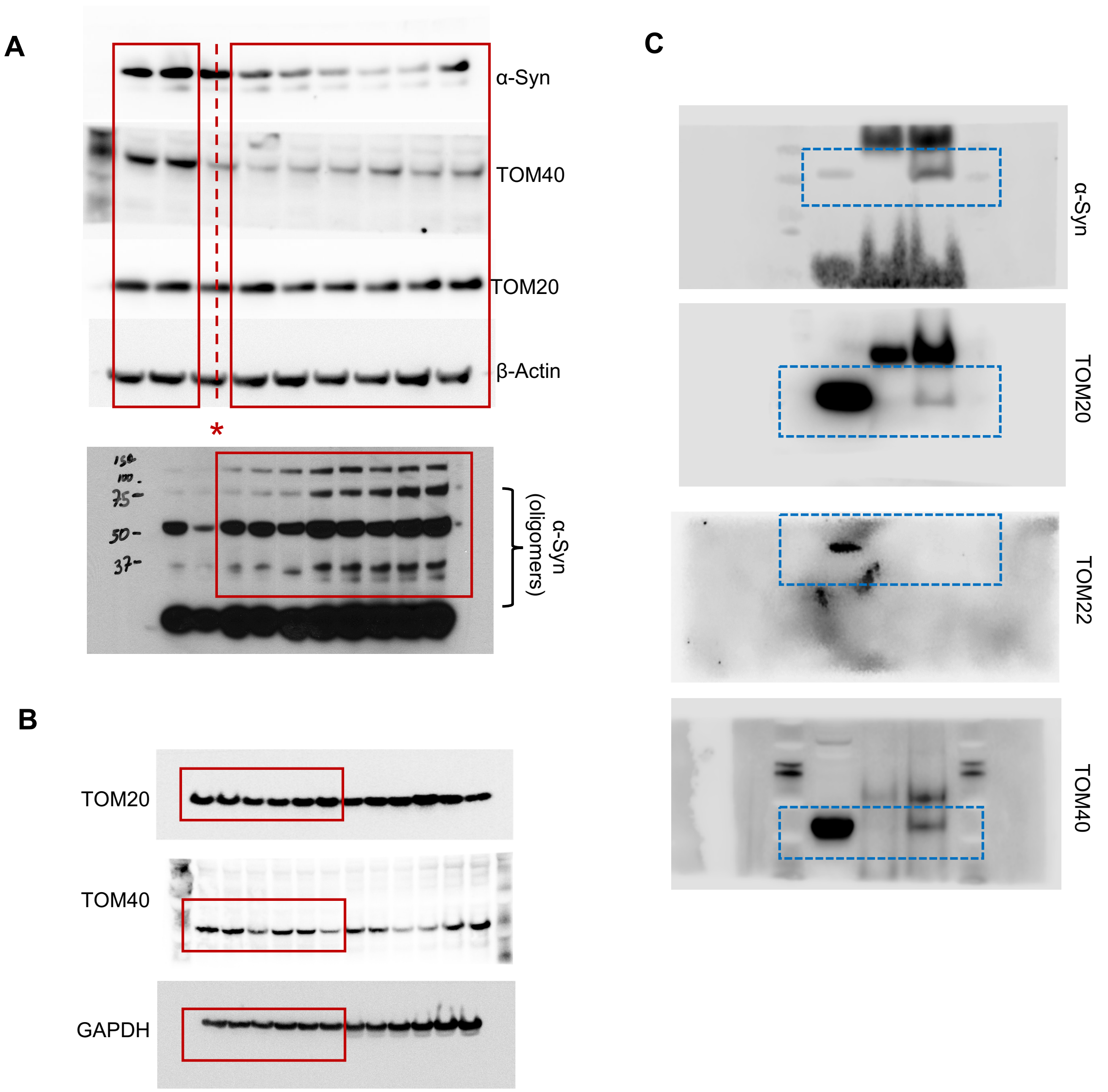

Supplementary File 2. A. Original Western Blot images for Fig. 2A. This figure presents a Western blot analysis wherein a specific lane, marked by a red star, has been subjected to splicing. **B**. Original Western Blot images for Fig. 2C. **C** Original Western Blot images for Supplementary Fig. 2B. The red box delineates the lanes selected for inclusion in the final representative image. The dashed blue box delineates the protein band of interest.

Supplementary File 3

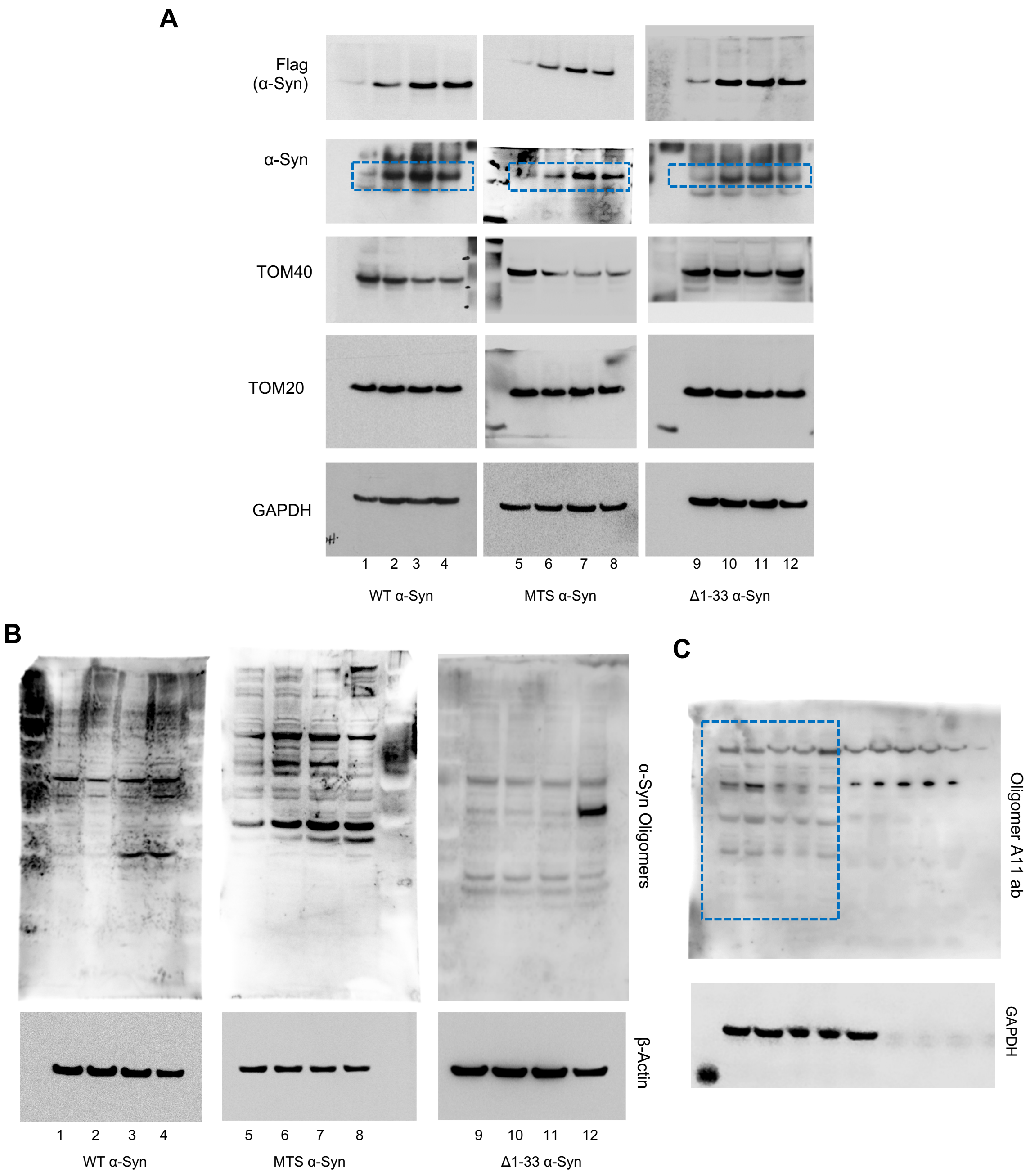

Supplementary File 3. A. Original Western Blot images for Fig. 3C. **B.** Original Western Blot images for Supplementary Fig. 3B. **C**. Original Western Blot images for Supplementary Fig. 3C. The dashed blue box delineates the protein band of interest.

Supplementary File 4

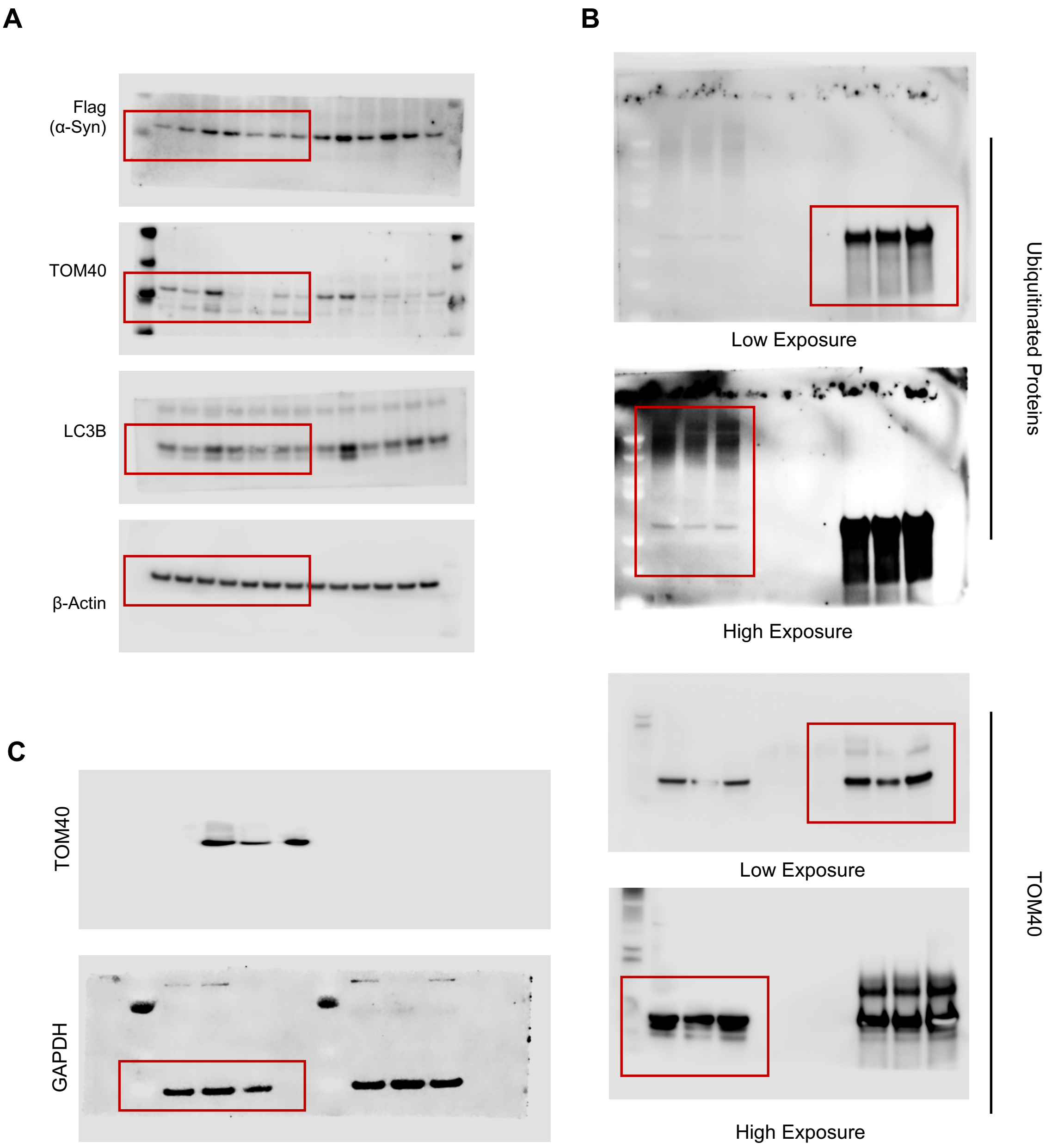

Supplementary File 4. A. Original Western Blot images for Fig. 4B. **B**. Original Western Blot images for Fig. 4E. **C**. Original Western Blot images for Fig. 6D. The red box delineates the lanes selected for inclusion in the final representative image.

Supplementary File 5

A

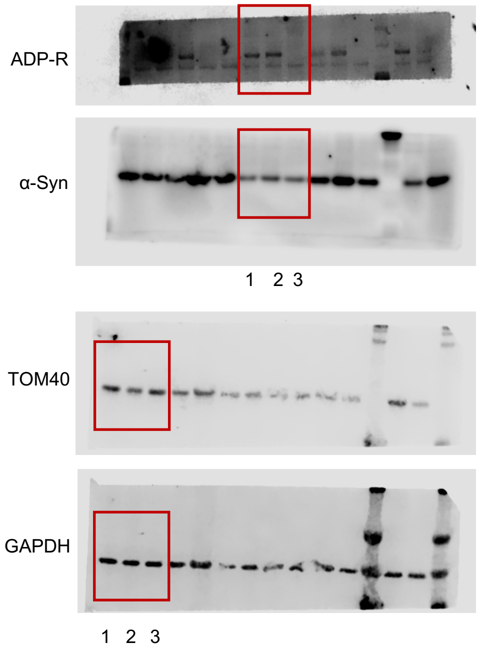

Supplementary File 5. A. Original Western Blot images for Fig. 7A. The red box delineates the lanes selected for inclusion in the final representative image.

Supplementary File 6. Original Agarose gel images and quantification data for Fig. 5 A. The red box delineates the lanes selected for inclusion in the final representative image**.**

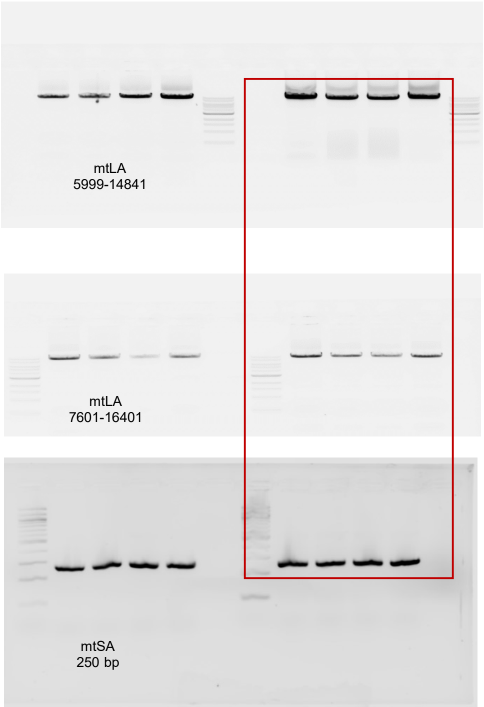

	Picogreen Fluorescence Units											
	Dox (-)			WT α-Syn			MTS α-Syn			Δ1-33 α-Syn		
mtLA 5999-14841	51743	50114	48196	45890	45757	45932	38917	35432	36471	47279	48727	49793
mtLA 7601-16401	41509	41804	37491	35248	36987	35265	31528	34381	37468	37918	36257	37291

	Fold Change											
	Ctrl α-Syn			WT α-Syn			MTS α-Syn			Δ1-33 α-Syn		
mtLA 5999-14841	1	1	1	0.917476	0.914817	0.918316	0.778065	0.70839	0.72916	0.945246	0.974196	0.995508
mtLA 7601-16401	1	1	1	0.875335	0.918521	0.875757	0.782954	0.853805	0.93047	0.941641	0.925226	0.92607

Supplementary File 7. Original Agarose gel images and quantification data for Fig. 5 B. The red box delineates the lanes selected for inclusion in the final representative image.

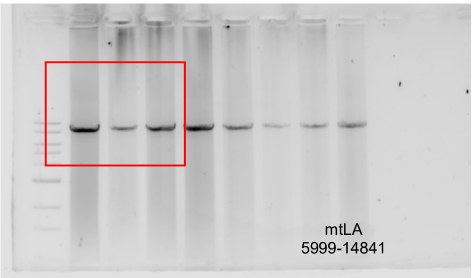

	Fold Change								
	Dox (-)			Dox (+)			Dox (+) & TOM40 OE		
mtLA 5999-14841	50122	52891	53503	23273	22973	26278	41731	45084	43511
mtLA 7601-16401	50758	50594	50709	26961	25095	24929	41150	46396	47457

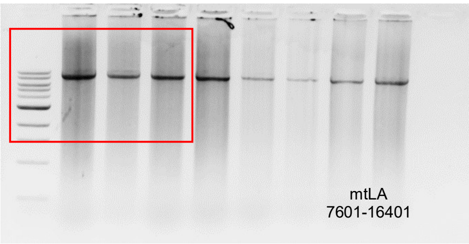

	Fold Change								
	Dox (-)			Dox (+)			Dox (+) & TOM40 OE		
mtLA 5999-14841	1	1	1	0.448608	0.442884	0.505943	0.800786	0.864761	0.834748
mtLA 7601-16401	1	1	1	0.534108	0.497467	0.494207	0.812728	0.91574	0.936575

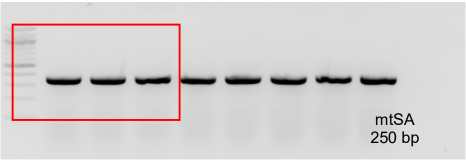

Supplementary File 8. Original Agarose gel images and quantification data for Fig. 5 F. The red box delineates the lanes selected for inclusion in the final representative image.

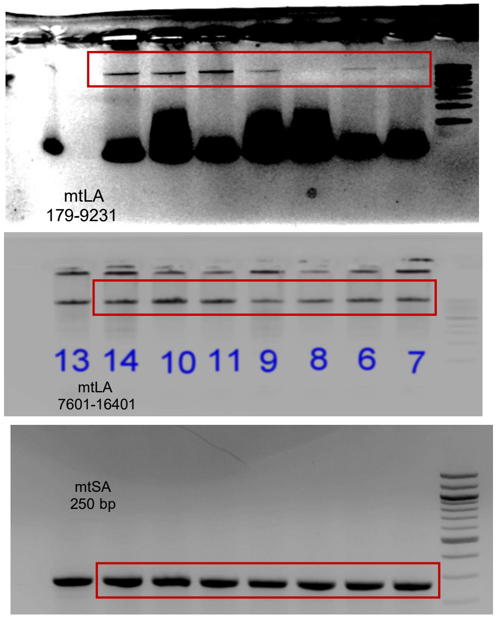

	Densitometry Analysis Values						
	Guam Control			Guam PD			
mtLA 179-9231	1	1	1	0.363209	0.057861	0.237763	0.071961
mtLA 7601-16401	1	1	1	0.468182	0.577273	0.727273	0.659091
